# Kinetics of Submerged Intersystem Crossings in Strongly Coupled Ion–Molecule Reactions: I^+^ + CH_3_X (X = F, Cl, Br, I)

**DOI:** 10.1021/acs.jpca.6c00949

**Published:** 2026-06-09

**Authors:** Tucker W. R. Lewis, Albert A. Viggiano, Shaun G. Ard, Nicholas S. Shuman

**Affiliations:** Air Force Research Laboratory, Space Vehicles Directorate, Kirtland Air Force Base, Albuquerque, New Mexico 87107, United States

## Abstract

The kinetics of the reaction between I^+^ and the methyl halides CH_3_X (X = F, Cl, Br, I) are measured at temperatures ranging from 300 to 600 K using a selected-ion flow tube apparatus. Exothermic product channels for X = F, Cl, Br forming CH_2_I^+^ + HX or CH_2_X^+^ + HI require an intersystem crossing, making the series a model system for the kinetics involving consistently large spin–orbit coupling. The reaction efficiencies relative to capture rates increase steeply along the series (X = F: 0.07, Cl: 0.22, Br: 0.67, I: 0.95); however, this is shown not to be a function of the halide mass or increasing coupling. Nonadiabatic transition state theory was applied to reaction coordinates calculated using density functional theory by treating minimum energy crossing points between singlet and triplet surfaces as proxies for the adiabatic transition states. This quantitatively reproduced both the magnitude and temperature dependence of the X = F, Cl, and Br reactions. The reaction efficiencies are controlled by the energy of the adiabatic transition state as the incident I^+^ approaches a hydrogen atom, leading to abstraction. The energies of those transition states are, in turn, a function of the entrance well depth of the triplet I^+^(H_3_CX) complexes, which are electrostatically bound and scale with the polarizabilities of the methyl halides. Charge transfer processes (dominating the X = I reaction and a minor product for X = Br) behave nonstatistically.

## Graphical abstract

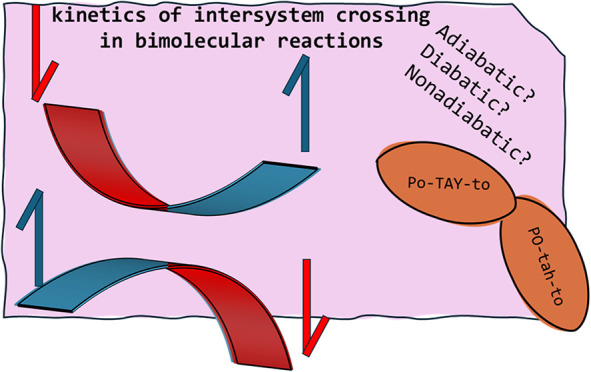

## Introduction

The terminology surrounding “adiabatic” and its derivatives is acknowledged as muddled, with the IUPAC Gold Book stating that when used the intended meaning should be explicitly stated.^1^ The direct translation of the etymological Greek root “diabainein” is “passable” (diabatic) modified to “impassable” (adiabatic). The modern scientific usage began with Scottish polymath William Rankine in the mid-19th century coining the thermodynamic meaning “with/without exchange of heat”;^2^ i.e., an adiabatic process occurs on a time scale much faster than that of heat exchange. The term hopped to quantum mechanics in the early 20th century with the invocation of the adiabatic theorem^3^ positing that under infinitely small perturbations, a system will not change eigenstate; i.e., the time scale of electronic relaxation is much faster than that of nuclear motion. Sensibly, a potential surface followed in that manner is termed the adiabat (adiabatic surfaces of like symmetry will never cross, coincidentally harkening back to the Greek root: impassable!). Counterintuitively, an adiabatic surface does not necessarily conserve characteristics such as electronic state. The converse, a diabatic state, is generally selected to conserve a particular characteristic, but is not an eigenstate of the full Hamiltonian, instead being an eigenstate of a reduced Hamiltonian omitting chosen term(s), e.g., spin–orbit coupling (SOC). Intuitively an adiabatic process is one proceeding on a single adiabat, but for a reaction undergoing an intersystem crossing (ISC; i.e., a nonradiative electron spin-flip) the jargon hits a morass of double-negation and polysemy. In this case, the adiabatic surfaces transition from one spin state to another, such that following the adiabat is undergoing an ISC, which is generally termed a nonadiabatic process! The terminology is not ideal, but we seem bound to it by the inertia of history. For clarity, at an ISC a diabatic transition conserves spin while an adiabatic transition changes spin. We emphasize that the key quality of the evolution of a reaction along a surface with either constant or varying total electron spin are the relative time scales of nuclear and electronic motion, as illustrated in Figure 1.

**Figure 1:**
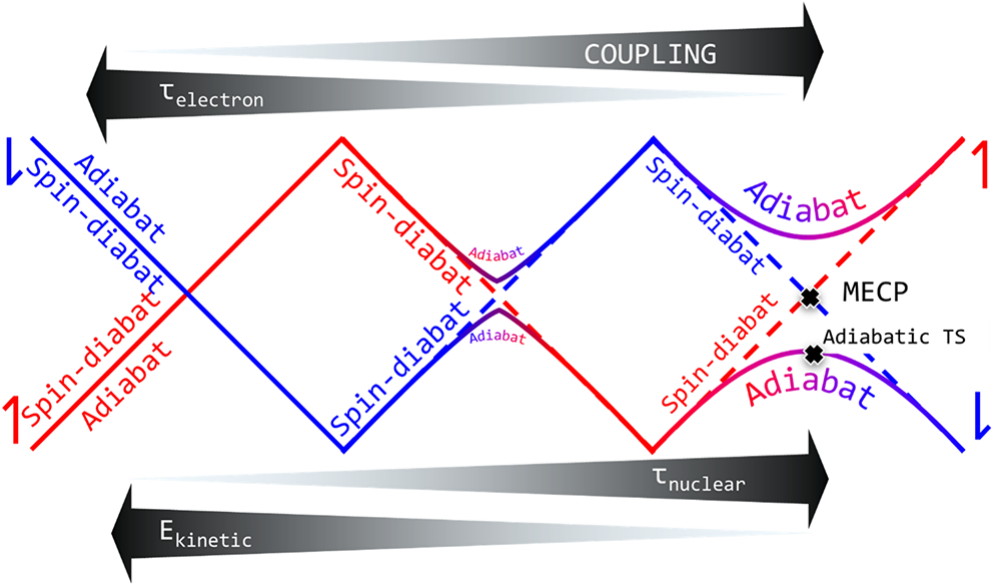
Schematic representation of the crossing of two surfaces of unlike spin indicating (left) the convergence of adiabatic and diabatic representations at the limits of large time scale for electronic motion (τ_electron_) driven primarily by small coupling and small time scale for nuclear motion (τ_nuclear_) driven primarily by large kinetic energy and (right) the divergence of the two representations at the opposite limits along with the divergence of the adiabatic transition state and the minimum energy crossing point. MECP indicates the minimum energy crossing point of the diabatic surfaces; TS indicates the transition state on the adiabatic surface.

The possible time scale for ISC events varies over many orders of magnitude, from subpicosecond to time scales limited only by those of the competing processes. While only the faster end of ISC can affect most photochemical processes,^4^ chemical reactions, particularly those involving ionic species, often involve long-lived intermediates on the nanosecond or longer time scales allowing even inefficient ISC to occur.^5^ This results in a prevalence of “spin-forbidden” ion–molecule reactions^6^ and discussion of “two-state” or “multi-state” reactivity in the literature.^7^ The consequences of ISC in ion–molecule reactions are variable: in some systems ISC is a rate-limiting bottleneck reducing reactivity,^8^ while in others ISC allows for otherwise inaccessible products increasing reactivity.^9^

As the time scale for electronic transition between states of differing multiplicity decreases relative to the time scale of nuclear motion (Figure 1), the adiabatic surfaces deviate increasingly from the spin-diabats and spin becomes a decreasingly “good” quantum number. The adiabatic transition state^10,11^ differs from the minimum energy crossing point (MECP) between the spin-diabats (in energy and possibly in geometry as well) and the excited adiabat becomes less involved in the reaction. It is tempting to ignore the spin-diabatic representations entirely in such cases, but studies of a number of presumably strongly coupled ion–molecule reactions show a distinct preference for spin-conserving reactivity even when a spin-changing channel is energetically preferred.^12–20^ This hints at a possible entropic preference for spin-conserved processes in ion–molecule reactions, even in strongly coupled systems where spin-conservation is not expected and has little obvious physical meaning. Additionally, calculation of an adiabatic transition state at an ISC is more challenging than that of a spin-conserving barrier. Most ion–molecule systems studied regarding ISC involve transition metal cations and as a result involve the electronic complexity of d or f orbitals. Here we explore a series of strongly coupled main group ion–molecule reactions with product channels that require ISC, with the overarching question of whether and how the kinetics are affected.

The series of methyl halides (CH_3_X, X = F, Cl, Br, I) have been used in many contexts as model systems for exploring fundamental aspects of chemical reactivity.^21–27^ While the equilibrium structures and the nature of bonding are similar across the series, many other characteristics vary smoothly. The C-X (X = F, Cl, Br, I) bond dissociation energies (BDE) are 4.7, 3.6, 3.0, 2.4 eV,^28^ respectively; the C–H BDE vary little 4.39, 4.34, 4.41, 4.47 eV;^29^ the isotropic polarizabilities are 2.62, 4.44, 5.55, 7.34 Å^3^;^30^ and the mass increases along with relativistic effects such as spin–orbit coupling. These qualities allow for extracting mechanistic insight from correlation, or lack thereof, with reactivity.

There is no expectation that the I^+^ + CH_3_X reactions should be similar to the well-studied X^–^ + CH_3_X reactions that are prototypical S_N_2 systems.^23,31–33^ Instead, we expect more similarity to transition metal cation reactions M^+^ + CH_3_X, which similarly involve ISC.^24,25,34^ In studying such systems, Armentrout and co-workers have shown a predominance for the reaction to follow a single adiabat at low kinetic energies and an increasing probability for a nonadiabatic transition to occur at large kinetic energies.

That behavior is explained using the Landau–Zener formalism.^35^ In ion–molecule reactions, the relatively strong ion-dipole or ion-induced dipole interactions result in correspondingly deeply bound entrance complexes. Subsequent rate-limiting features of the potential surface are often submerged (i.e., at lower energy than reactants) leading to behaviors qualitatively distinct from neutral systems. Neutral bimolecular reactions typically must overcome a positive energetic barrier, while ion–molecule reactions with barrierless entrance wells and submerged transition states often have rate constants with a negative temperature dependence owing to the entropic preference of the former over the latter. Most kinetic treatments of ISC are for neutral systems, approaching the crossing seam “from below” such that tunneling can be of import and excess energy is always small. Submerged ISC should show distinct kinetics, as has been invoked to explain observed behavior in ion–molecule systems previously, for instance see the discussion in Rue et al.^36^ Briefly, for weakly coupled systems the Landau–Zener formalism for behavior at an avoided crossing is

1 $$P_{\mathrm{LZ}}=e^{-2\pi H_{12}^{2}/\hbar \left|\frac{\delta E}{\delta r}\right|{\left(\frac{E-E_{c}}{\mu_{m}}\right)}^{1/2}}$$

where *P*_LZ_ is the probability of remaining on the diabat, *H*_12_ is the nonadiabatic coupling strength, $\frac{\delta E}{\delta r}$ is the change in potential energy along the reaction coordinate, *E* is the energy of the system relative to reactants, *E*_*c*_ is the energy of the crossing seam relative to reactants, and μ_*m*_ is the reduced mass, such that *E – E*_*c*_ is the kinetic energy of the system and ${\left(\frac{E-E_{c}}{\mu_{m}}\right)}^{1/2}$ is the nuclear velocity. Equation 1 suggests an *E* ^–1/2^ behavior in the crossing probability when *E* ≫ *E*_*c*_ but little dependence on *E* when *E < E*_*c*_. Indeed, this behavior describes well the measured cross sections as a function of energy for the spin-forbidden ^4^Zr^+^ + ^1^CO_2_ → ^2^ZrO^+^ + ^1^CO reaction (where the superscripts indicate multiplicity).^37^ For strongly coupled system, the Zhu-Nakamura^38^ formalism is more appropriate, but both that and *P*_LZ_ will approach 0, i.e., the reaction will not deviate from the ground state adiabat.

While weakly coupled ion–molecule reactions show distinct kinetics, in this limit of large coupling strength, where the reaction must proceed over the submerged adiabatic transition state, should the kinetics differ from those of a reaction with a submerged transition state in a system devoid of ISC?

In the present study we report temperature-dependent kinetics of I^+^ + CH_3_X (X = F, Cl, Br, I). Through a combination of quantum chemical calculations and statistical modeling, we unravel the reaction mechanisms. Where ISC is required, we evaluate whether the behavior differs from ion–molecule reactions that do not involve ISC.

## Methods

### Experimental Section

The variable ion source temperature adjustable selected-ion flow tube (VISTA-SIFT) apparatus (Figure 2) used for these experiments has been detailed previously and only a brief description will be provided here.^39,40^ I^+^ was produced in a flowing afterglow ion source, similar in design to other apparatuses described elsewhere.^41^ A fast flow of He (Matheson 99.999%) flowed through a 1” diameter quartz tube, expanded into a 2.5” diameter stainless steel flow tube, and evacuated using by a Dry Screw Pump (10^4^ L s^–1^) prior to a nosecone with a 2 mm aperture. An Evenson style cavity was used to ignite a microwave discharge in the flow,^42^ producing He^+^, He_2_^+^, metastable He*, and e^–^. Twenty-five cm downstream a second inlet introduced a very small flow (≪1 std. cm^3^ min^–1^) of CF_3_I (Sigma-Aldrich) using a variable leak valve (Granville-Phillips). I^+^ was produced presumably through dissociative ionization with He^+^ and dissociative Penning ionization with He*. I^+^ reacts rapidly with CF_3_I,^43^ requiring that the concentration of CF_3_I be kept low and neither the He^+^ nor He* will have been depleted. Small amounts of I_2_^+^ (∼0.5%) and IO^+^ (∼5%) were also produced. After extraction through the nosecone the ions were transported using a pair of rectilinear ion-guides^44^ to the entrance of a quadrupole mass filter. I^+^ was mass-selected and transported through a series of trapezoidal quadrupole ion-guides^44^ to gradually reduce the size of the trapping potential and injected via a Venturi inlet situated into a 3.5 cm diameter flow tube maintained at ∼0.35 Torr with a fast flow of helium. Ions underwent on the order of 10^4^ collisions with He to thermalize to the wall temperature. At the terminus of the flow tube, ions were extracted through a rounded, carbon-coated nose cone, through a rectilinear ion-guide to an orthogonally accelerated Reflectron time-of-flight mass spectrometer (Jordan, TOF-MS) where they were detected and counted using a Z-stack of microchannel plates. To measure the reactivity of the I^+^ ions a metered flow of CH_3_X (X = F, Cl, Br, I) was introduced through a 1/8” diameter molybdenum finger inlet located 59 cm prior to the terminus of the flow tube. Both reactant and product ions were detected using the TOF-MS. Pseudo-first order rate constants were obtained by monitoring the relative abundance of the reactant ion as a function of the much larger CH_3_X concentration. The temperature of the flow tube was varied using resistive heating elements surrounding the flow tube.

**Figure 2:**
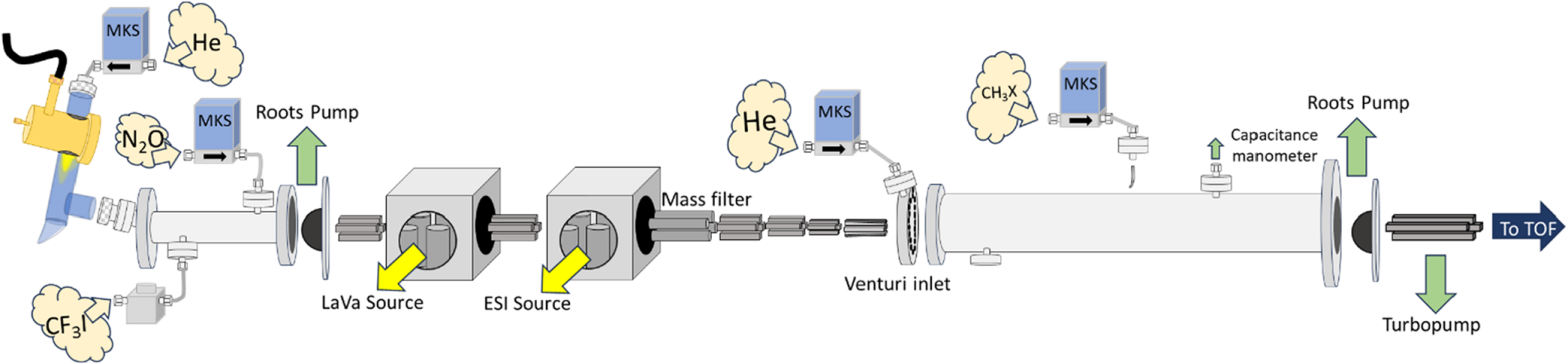
Cartoon schematic of the VISTA-SIFT apparatus with flowing afterglow ion source as used in the present experiment.

I^+^ has a ^3^P_2_ ground state with large spin–orbit splittings to ^3^P_0_ and ^3^P_1_ states (about 0.80 and 0.88 eV, respectively). The first excited singlet state ^1^D_2_ lies 1.70 eV above the ground state.^45^ These large energy gaps resulted in a low efficiency of quenching for collisions with the helium buffer gas. In prior work using a distinct SIFT apparatus with similar quenching conditions, excited electronic state(s) of I^+^ persisted throughout the ms time scale of the experiment.^46^ Ground state I^+^ was identified to react slowly (*k* = 4.5 × 10^–11^ cm^3^ s^–1^) with Cl_2_, while excited state species (presumed to be the triplet spin–orbit states) reacted with a rate constant about 1 order of magnitude larger. Here, reaction with Cl_2_ was used as a monitor for the excited state population. I^+^ produced in the flowing afterglow source either through He^+^/He* + CF_3_I or Ar^+^ + CF_3_I decayed biexponentially with Cl_2_, consistent with the literature rate constants. Addition of a polyatomic quenching gas into the source flow tube eliminated the faster component of the decay. CH_4_ was effective at 10 std. cm^3^ min^–1^ but produced HI^+^ complicating mass-selection. Instead, N_2_O quenchant was used, despite being less effective, requiring 100 std. cm^3^ min^–1^.

Additionally, reaction of the I^+^ with O_2_ produced O_2_^+^. The charge transfer reaction is 1.65 eV endothermic from the ground state, but close to resonant with the first excited I^+^ singlet state. Surprisingly, addition of O_2_ to source flow tube did not affect the observation of O_2_^+^ in the reactant flow tube, suggesting that the singlet excited state was produced instead upon injection of I^+^ into the flow tube. The excited state remained present at 1–3% of the ground state. As a result, products observed at reaction efficiencies of less than about 5% of the capture rate constants (following the Su-Chesnavich^47^ parametrized values) must be considered with extra scrutiny.

### Quantum Chemical Calculations

Stationary points along the reaction coordinates for reactions 1–4 were calculated using the ORCA 6.0.1 quantum chemical software.^48,49^ Geometries were optimized at the B3LYP/x2c-TZVPPall^50^ level of theory with the RIJCOSX approximation and the exact 2-component (X2C)^51^ relativistic Hamiltonian including spin–orbit coupling. Minima and transition states were confirmed to have 0 or 1 imaginary frequencies respectively under normal-mode analysis. Transition states were confirmed to connect to adjacent minima via intrinsic reaction coordinate (IRC) calculations. MECP at this level were found using the algorithm detailed by Harvey et al.^52^ as implemented in ORCA. MECP are treated in the statistical analysis as pseudotransition states, extracting vibrational frequencies via a normal-mode analysis, discarding the mode corresponding most closely to the reaction coordinate. Spin orbit coupling constants were calculated using time-dependent density functional theory (TD-DFT) at the B3LYP/x2c-TZVPPall level with the X2C Hamiltonian with spin–orbit coupling. Results are shown in Figures S1–S4.

Zero-point corrected energies were compared to 13 reactions of the form I^+^ + CH_3_X for which literature thermicities are well-known^28^ (see SI). The calculations show a bias being an average of 0.35 eV more exothermic than the literature, but the errors are surprisingly consistent about that value with a standard deviation of just 0.1 eV. The spin–orbit averaged I^+^ energy is 0.38 eV above the ^3^P_2_ ground state, possibly explaining the 0.35 eV offset under the assumption that the spin–orbit averaged energies of the reaction intermediates and products are closer to their ground states. All calculated energies at this level presented in the main text have been adjusted by 0.35 eV relative to reactants. The 2σ uncertainty in the reported calculated energies is estimated at ± 0.25 eV. Optimized geometries, energies, and vibrational frequencies are provided in Table S3.

### Statistical Modeling

Details of a statistical modeling approach to ion–molecule reactions have been provided elsewhere.^53^ Briefly, kinetics were determined by stochastically running trials each with initial conditions (collision energy, internal energies, impact parameter) selected from thermal distributions. Complex formation was assumed to be controlled by a long-range electrostatic potential; trials with collision energy greater than the maximum along that potential proceeded to entrance complexes while those with lower collision energies were nonreactive. The methodology reasonably reproduces the capture rates following the parametrization of Su and Chesnavich.^47^ Specific unimolecular rate constants *k*(E,J)^54,55^ were calculated for each isomerization and dissociation from each stationary point along reaction coordinates shown in Figure 2. A trial proceeded probabilistically from the entrance complex according to *k*(E,J) for all possible channels while also competing with energy transfer to or from a buffer gas through third-body collisions.^56^ A trial continued until either a dissociation occurred or a complex was stabilized to an energy below all isomerization and dissociation thresholds so that leaving the complex was unlikely.

This stochastic implementation of transition state theory (TST) is modified here to be a stochastic implementation of nonadiabatic transition state theory (NA-TST) by accounting for the probability of adiabatic versus diabatic behavior at intersystem crossing points. Intersystem crossing was handled following the methodology described by Harvey et al.^57^ An MECP is treated as a transition state by calculating the number of states excluding the normal mode most closely following the reaction coordinate. Additionally, the Landau–Zener (L–Z) or Zhu-Nakamura (Z–N) nonadiabatic transition probability was calculated using spin–orbit coupling constants shown in Figures S1–S4. In principle, this allows for a transition to an excited adiabat which reduces the likelihood of isomerization along the ground state adiabat; however, in practice for the systems here, the calculated diabatic transition probability was effectively zero under all relevant conditions. That is to say, while an NA-TST approach was implemented, the nonadiabatic component was found not to be needed to reproduce the experimental data for these systems.

### Statistical Kinetic Analysis

Partial rate constant distributions were obtained by applying the Statistical Kinetic Analysis (SKA) method described elsewhere^58^ and summarized here. A best fit to the data was obtained by first defining a system of chemical reactions to produce the observed products. This system of reactions is converted into a system of ordinary differential equations (ODEs) that are then integrated for a given set of rate constants and initial concentrations using a Dormand-Prince 5(4) solver. This yields a concentration matrix for all species as a function of CH_3_X concentration that can be compared to the measured values to yield a goodness-of-fit (GOF). This GOF describes the difference between the data and the concentration matrix for the given set of rate constants and initial concentrations. The GOF is minimized with the differential evolution algorithm to stochastically sample different initial concentrations and rate constants to find the set of values that yields a best fit. Once a best fit is obtained the GOF, rate constants, and initial concentrations are recorded. To determine the uncertainty of the fit a bootstrapping resampling method is employed. The measured data is resampled with replacement to yield a new sample of the data. The sample is fit using the same procedure and the new GOF, rate constants, and initial concentrations recorded for the sample. This process is repeated many times (500 here) to build up a population for each partial rate constant.

The final distributions for each partial rate constant were found by applying a correction factor to the measured rate constants. The correction factor was obtained by taking several measurements of a reaction with a known rate constant believed to go at or very near the collision limit, Ar^+^ + CH_4_. From this measurement the rate constant value and the standard error were obtained. The ratio of the measured rate constant to its literature value (with its own standard error) was taken by drawing 1 × 10^5^ samples from each distribution and taking the ratio of these samples to build up the resulting distribution of the correction factors. The partial rate constants from SKA were convoluted with the correction factors by a similar method: 1 × 10^5^ samples were taken from the correction factors and each was multiplied by the partial rate constants from a trial. This preserved the ratio of the rate constants to each other while taking into account that the full trial could be biased.

The total rate constant for each reaction was obtained by summing up the corrected partial rate constants from each bootstrapping sample. Product branching ratios were obtained by dividing the corrected partial rate constant distributions by the corrected total rate constant distributions in a manner similar to those described above.

## Results

### Reaction Coordinates

Stationary points along reaction coordinates calculated at the B3LYP/x2c-TZVPPall level are shown in Figure 3 for reactions 1–4. All four coordinates share qualitative similarities. The reactant surfaces are of triplet multiplicity and can form two entrance complex geometries, corresponding to “X-approach” or “H-approach” of the incident I^+^. Both structures are primarily electrostatically bound, with the strength of the attraction increasing with polarizability from X = F to I. Similarly, the excited state reactant singlet surfaces have wells corresponding to “X-approach” or “H-approach”. In these cases, covalent I–X bonds are formed resulting in deeper wells on “X-approach”. “H-approach” leads directly to hydrogen abstraction (HA) and HI + CH_2_X^+^ products. Hydrogen abstraction by the I atom is also possible from the “X-approach” intermediate but requires isomerization over a substantial barrier. Alternatively, a methyl migration exchanges the X and I atoms and can lead to hydrogen abstraction by the X atom and the energetically preferred products HX + CH_2_I^+^ but also over a substantial barrier.

**Figure 3:**
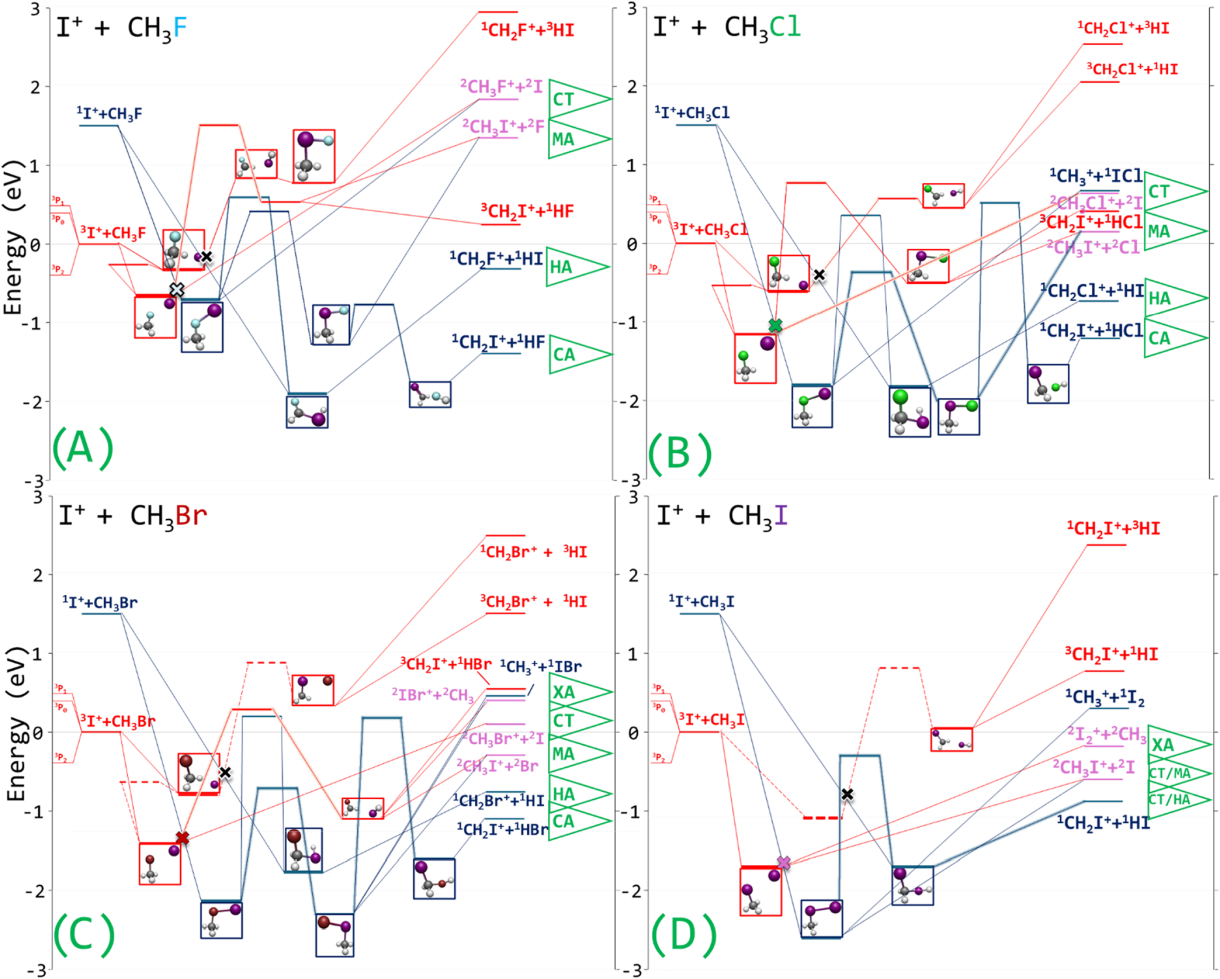
Stationary points along reaction coordinates for I^+^ + CH_3_X ((A) X = F; (B) X = Cl; (C) X = Br; (D) X = I) as indicated calculated at the B3LYP/x2c-TZVPPall level. Multiplicity (red, triplet; blue, singlet; purple, triplet or singlet) is indicated by color; varying shades are only to help the eye. Energies are zero-point corrected and relative to separated reactants. Energies and approximate locations of minimum energy crossing points, determined as described in the text, are indicated by x’s. Two-letter codes on the right axis refer to product channels as defined in the text and indicate the thermicities (hydride abstraction, HA; carbene abstraction, CA; methyl abstraction, MA; halogen abstraction, XA; charge transfer, CT).

Both HI + CH_2_X^+^ and HX + CH_2_I^+^ ground state products are singlets and formally spin-forbidden products, with spin-allowed excited state products being endothermic. In the cases of X = F and X = Cl, these are the only energetically accessible product channels. For X = Br, spin-allowed Br + CH_3_I^+^ products are slightly exothermic and the charge transfer reaction yielding I + CH_3_Br^+^ is endothermic by less than 0.1 eV. For X = I, HI + CH_2_I^+^ is calculated to be slightly energetically preferred over the exothermic I + CH_3_I^+^ charge transfer, while the I_2_^+^ + CH_3_ channel is also exothermic, but less so.

In all cases, the energetically preferred product requires ISC to a singlet surface. Two distinct crossing regions are identified: one near the bottom of the entrance well to the triplet “X-approach” complex and a second near the “H-approach” entrance well. The former can lead to products only through further isomerization, while the latter crossing leads to direct hydrogen abstraction by the I atom.

### Kinetics

Measured total and partial rate constants are shown in Figure 4 and Tables 1 and 2. The total rate constants increase by about an order of magnitude from X = F to I. At the same time, the capture rate constants^47^ decrease from 2.0 × 10^–9^ cm^3^ s^–1^ at 300 K for X = F to 1.1 × 10^–9^ cm^3^ s^–1^ at 300 K for X = I, such that the reaction efficiency increases from about 0.05 for X = F up to 1.0 for X = I.

**Figure 4:**
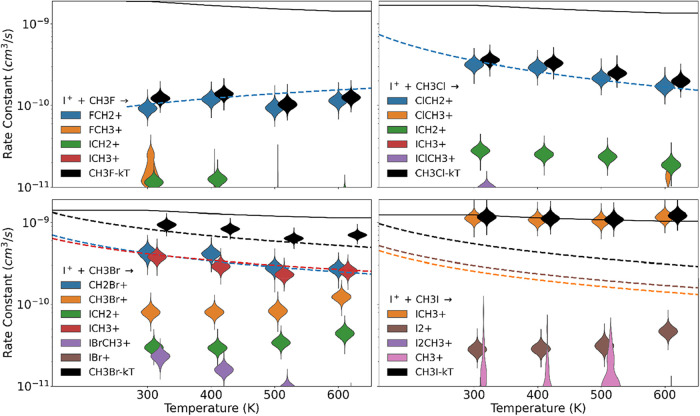
Violin plots of measured rate constants for reactions 1–4. The legend label indicates the reaction, with the different colors giving the partial rate constant leading to formation of the given product. The largest valued violin plot in each panel is the total rate constant (black), denoted by “–k_T_”. The solid black line is the collision rate given by the parametrized Su-Chesnavich value.^47^ The dashed colored lines are the statistically modeled best-fits. The violin plots represent the SKA^58^ fit rate constant distributions for each partial rate constant. Taller violin plots represent less well determined rate constants than shorter ones. The widest point in each violin is the most probable value.

**Table 1:** Total Rate Constants as a Function of Temperature with Asymmetric Uncertainties (±) Determined as Described in the Text^a^ ^,^ ^b^

Species	*T*(K)	*k* (×10^–10^ cm^3^ s^–1^)	*k*_capture_ (×10^–10^ cm^3^ s^–1^)	Efficiency
I^+^ + CH_3_F	300	1.24 (+0.67/–0.44)	18.8	0.07 (+0.04/–0.02)
	400	1.41 (+0.78/–0.54)	16.7	0.08 (+0.05/–0.03)
	500	1.05 (+0.73/–0.42)	15.2	0.07 (+0.05/–0.03)
	600	1.27 (+0.76/–0.48)	14.2	0.09 (+0.05/–0.03)
I^+^ + CH_3_Cl	300	3.63 (+1.80/–1.28)	16.8	0.22 (+0.11/–0.08)
	400	3.32 (+1.51/–1.29)	15.4	0.22 (+0.10/–0.08)
	500	2.51 (+1.20/–0.80)	14.3	0.18 (+0.08/–0.06)
	600	2.01 (+1.63/–0.72)	13.5	0.15 (+0.12/–0.05)
I^+^ + CH_3_Br	300	9.40 (+3.46/–2.86)	14	0.67 (+0.25/–0.20)
	400	8.38 (+2.88/–2.08)	12.8	0.65 (+0.22/–0.16)
	500	6.40 (+2.19/–1.65)	12	0.53 (+0.18/–0.14)
	600	7.04 (+2.08/–1.81)	11.4	0.62 (+0.18/–0.16)
I^+^ + CH_3_I	300	11.77 (+6.34/–4.20)	12.4	0.95 (+0.51/–0.34)
	400	11.26 (+6.98/–3.96)	11.4	0.99 (+0.61/–0.35)
	500	10.94 (+6.46/–4.22)	10.8	1.01 (+0.60/–0.39)
	600	12.26 (+7.12/–4.77)	10.4	1.18 (+0.68/–0.46)

a Efficiency is the rate constant divided by the capture rate.

b Ref 47.

**Table 2:** I^+^ + CH_3_X Product Branching Fractions at 300 K^a^

Reaction	Observed Product	Reaction Type	Branching fraction	Thermicity (eV)
I^+^ + CH_3_F	CH_2_F^+^	HA	0.88 (+0.14/ – 0.21)	–0.2
	CH_2_I^+^	CA	0.12 (+0.11/–0.06)	–1.3
I^+^ + CH_3_Cl	CH_2_Cl^+^	HA	0.88 (+0.05/–0.08)	–0.52^b^
	CH_2_I^+^	CA	0.08 (+0.06/–0.04)	–1.1
	IClCH_3_^+^	AS	0.03 (+0.02/–0.01)	–2.2
I^+^ + CH_3_Br	CH_2_Br^+^	HA	0.45 (+0.15/–0.17)	–0.7
	CH_3_Br^+^	CT	0.09 (+0.07/–0.04)	0.09^b^
	CH_2_I^+^	CA	0.03 (+0.03/–0.01)	–1.1
	CH_3_I^+^	MA	0.40 (+0.18/–0.14)	–0.34^b^
	IBrCH_3_^+^	AS	0.02 (+0.02/–0.01)	–2.2
I^+^ + CH_3_I	CH_3_I^+^	CT/MA	0.98 (+0.02/–0.10)	–0.91^b^
	I_2_^+^	XA	0.02 (+0.02/–0.01)	–0.28^b^
	CH_3_I_2_^+^	AS	0.00 (+0.01/–0.00)	–2.7

a Thermicities are calculated at B3LYP/x2c-TZVPPall except where noted. Minor observed ions that are determined not to be from ground state reactants are excluded; see Table (S2).

b Derived from literature ref 28.

We generalize the observed product channels as

$$I^{+}+{\mathrm{CH}}_{3}\to {\mathrm{CH}}_{2}X^{+}+\mathrm{HI}\left(\mathrm{hydride abstraction},\mathrm{HA}\right)$$

$$\to {\mathrm{CH}}_{2}I^{+}+\mathrm{HX}\left(\mathrm{carbene abstraction},\mathrm{CA}\right)$$

$$\to \mathrm{CH}3I^{+}+X\left(\mathrm{methyl abstraction},\mathrm{MA}\right)$$

$$\to {\mathrm{IX}}^{+}+\mathrm{CH}3\left(\mathrm{halogen abstraction},\mathrm{XA}\right)$$

$$\to \mathrm{CH}3X^{+}+I\left(\mathrm{charge transfer},\mathrm{CT}\right)$$

$$\to \mathrm{CH}3{\mathrm{XI}}^{+}\left(\mathrm{association},\mathrm{AS}\right)$$

Reaction 1, I^+^ + CH_3_F, is dominated at all temperatures by the HA channel (1_HA_), production of CH_2_F^+^ + HI, with a possible minor contribution from the 1_CA_ channel (CH_2_I^+^ + HF). While CH_3_F^+^ is observed, formation is heavily endothermic (including from singlet I^+^) and is most likely due to a small current of He^+^ leaking through the mass filter.

I3++CH13F→CH12I++HF1+1.3eV 1CA

→CH12F++HI1+0.2eV 1HA

The dominant HA channel is energetically dispreferred to CA (exothermic channels are listed here with 0 K thermicities from literature where uncertainties (2σ) are indicated or as calculated at the B3LYP/x2c-TZVPPall level where they are not; endothermic channels are detailed in the SI). Both are formally spin-forbidden. The spin-allowed triplet 1_HA_ channels are calculated >2.5 eV endothermic and cannot be contributing from ground state reactants. The spin-allowed 1_CA_ (^3^CH_2_I^+^) channel is calculated at 0.1 eV endothermic and cannot be easily dismissed. While the total rate constant is nearly temperature-independent, the reaction efficiency shows a mild positive temperature dependence.

Reaction 2, I^+^ + CH_3_Cl, has about a three times larger total rate constant and four times higher efficiency than Reaction 1. Reaction 2 is also dominated by the spin-forbidden 2_HA_ (CH_2_Cl^+^ + HI) channel but with a larger (∼5%) contribution from the 2_CA_ (CH_2_I^+^ + HCl) channel. The spin-allowed triplet 2_CA_ channel is calculated to be 0.1 eV endothermic and could contribute.

I3++CH13Cl→CH12I++HCl1+1.1eV 2CA

→CH12Cl++HI1+0.52±0.003eV 2HA

Reaction 2 shows a mild negative temperature dependence in both the rate constant and efficiency.

Reaction 3, I^+^ + CH_3_Br, has additional energetically accessible product channels.

I3++CH13Br→CH12I++HBr1+1.1eV 3CA

→CH12Br++HI1+0.7⁡eV 3HA

→CH23I++Br2+0.335±0.003⁡eV 3MA

→CH23Br++I2−0.090±0.003eV 3CT

Both the total rate constant and the reaction efficiency exceed that of Reaction 2 and both show a small negative temperature dependence. Unlike Reactions 1 and 2, the 3_HA_ (CH_2_Br^+^ + HI) does not dominate the reaction as both the spin-forbidden 3_HA_ and spin-allowed 3_MA_ (CH_3_I^+^ + Br) channels contribute similarly. Both channels show similar small negative temperature dependences. Note that the MA channel does not occur via an S_N_2 mechanism. Instead, the incident I^+^ attacks the Br atom directly, and abstracts the methyl through a three-centered transition state. The 3_CT_ (CH_3_Br^+^ + I) channel is slightly endothermic, but is a minor channel contributing between 10–20% of all products and with a small positive temperature dependence in agreement with the endothermicity. Smaller contributions from 3_CA_ (CH_2_I^+^ + HBr) and 3_AS_ (ICH_3_Br^+^) are also seen.

Reaction 4, I^+^ + CH_3_I, occurs within uncertainty at the capture rate constant at all temperatures. All or nearly all product is from 4_CT_ (CH_3_I^+^ + I) with a possible minor contribution of 4_XA_ (I_2_^+^ + CH_3_). The most exothermic channel 4_CA/HA_ (CH_2_I^+^) is not observed.

I3++CH13I→CH12I++HI1+1.2eV 4CA/HA

→CH23I++I2+0.913±0.002eV 4CT/MA

→I22++CH23+0.276±0.001eV 4XA

Reaction 4 is the only of these systems that, to our knowledge, has been studied previously. One of the earliest mass spectrometric ion–molecule studies study reported I_2_^+^ product, possibly, but not definitively from reaction 4.^59^ Beauchamp et al. briefly mention observation of the 4_CT/MA_ reaction, but note that the I_2_^+^ 4_XA_ product was above the range of the mass spectrometer.^60^ Finally, a study in 1994 reported that I_2_^+^ was only formed from reaction of I^+^ with (CH_3_I)*_n_* clusters where *n* > 1.^61^

## Discussion

The kinetics measured across reactions 1–4 show trends in rate constant magnitude, reaction efficiency, product branching, and temperature dependence (Table 1). We aim to explain these trends using reaction coordinates derived from quantum chemical calculations and statistical modeling results. Ideally, we would correlate these results to trends in physical properties across the series of CH_3_X.

By inspection, the efficiency of the reactions increases markedly down the periodic table from X = F to I. As three of the four reactions make primarily spin-forbidden products, this could be interpreted as an increase in ISC due to the increasing masses and spin–orbit coupling; however, calculations at the TD-DFT level show substantial spin–orbit coupling for all four reactions (Figures S1–S4), such that LZ or ZN theory predict almost purely adiabatic behavior in all cases. Instead, we see below that energetics alone explains the reactivity.

For X = F, Cl, Br, the energetically preferred CA channel is heavily outcompeted by the HA channel. Inspection of the calculated reaction coordinates provides an explanation: the isomerization energy from the CH_3_I^+^(X) intermediate to the CH_2_I^+^(HX) exit complex is prohibitive. CT becomes significant in X = Br, despite the channel being barely exothermic and energetically dispreferred to other products, and CT is dominant in X = I where the channel is appreciably exothermic.

NA-TST assuming a transition state located at the calculated MECP reproduces the experimental data for the major products of X = F, Cl, Br, but fails at X = I (Figure 3). In all cases, the calculated SOC results in near zero probability of diabatic transitions occurring; that is, as modeled ISC always occurs at the MECP. The extent of the agreement is surprising, considering the limited expected accuracy of the calculations and the coarse approximation of treating the MECP structure as a transition state (an error in a rate-limiting transition state energy of just 0.1 eV will typically shift the modeled rate constant by about 1 order of magnitude).^53^ The DFT calculations do not incorporate spin–orbit coupling, which should be significant in these systems and would, to a first approximation, yield an adiabatic transition state at lower energy than the MECP. In fact, the best fits to the experiment shown in Figure 4 require a small positive adjustment of about 0.15 eV (+0.16, +0.13, and +0.18 eV, for X = F, Cl, Br, respectively) to the calculated MECP energies. In these three cases, TST accurately reproduces the magnitude and temperature dependences of the major products of each reaction. In all three cases, the modeled behavior is adiabatic; that is, ISC occurs at essentially every encounter of the MECP and consideration of diabatic transitions is unimportant. The reactions act similarly to typical, spin-conserving ion–molecule reactions.

For X = F, the magnitude and small positive temperature dependence of CH_2_F^+^ production (1 HA reaction) is well-reproduced assuming a transition state isoenergetic with reactants. The small observed quantity of CH_3_F^+^ (highly endothermic) must be due to the excited state I^+^ contaminant, while the source of the small observed quantity of CH_2_I^+^ is more ambiguous, but also likely due to excited state reactants.

For X = Cl, the magnitude and negative temperature dependence of the CH_2_Cl^+^ product (2_HA_ reaction) is reproduced assuming a transition state 0.25 eV lower in energy than separated reactants. The minor CH_2_I^+^ (2_CA_) channel is not reproduced, indicating that either the calculations overestimate the barriers along this pathway or that it arises from excited state contamination. The negative temperature dependence of the reaction suggests a process without barriers at energies above separated reactants, more in line with the source being an excited state.

For X = Br, the CH_2_Br^+^ product (3_HA_) is well-reproduced assuming a transition state 0.35 eV below separated reactants. The CH_3_I^+^ product (3_MA_) competes, with the transition state between I^+^(CH_3_Br) and Br^+^(CH_3_I) on the singlet surface being the rate-limiting step. TST reasonably reproduces the temperature dependences of the CH_3_Br^+^ (3_CT_) and CH_2_I^+^ (3_CA_) products but underestimates their magnitudes by about a factor of 10. The CH_2_I^+^ product (3_CA_) has a positive temperature dependence, suggesting at least a portion is formed through a near endothermic reaction (i.e., from ground state I^+^, not from excited state). The CH_3_Br^+^ product is sufficiently large (about 10% of the capture rate constant) that it cannot be attributed primarily to reaction from excited state I^+^. Instead, we suggest that this slightly endothermic (by 0.090 ± 0.003 eV) charge transfer channel does not proceed statistically. Finally, the experiment shows a slow association channel with a typical steep temperature dependence. TST does not predict a measurable amount of association, indicating an underestimation of the I^+^(CH_3_Br) binding energy.

For X = I, TST does not reproduce the data. The experiment shows a capture-controlled reaction producing exclusively CH_3_I^+^ (4_CT/MA_). TST predicts an efficient, but subcollisional reaction with competition between CH_3_I^+^ and CH_2_I^+^ (4_MA_) products. As in the Br reaction, the charge transfer reaction does not appear statistical. Instead, we suggest that there is a favorable curve crossing to charge transfer products early in the reaction coordinate, but at a distance smaller than that of the entrance well centrifugal barrier, such that the total rate remains capture-controlled. This interpretation suggests that the observed process is a charge transfer rather than a methyl abstraction, the latter needing to sample a longer lived intermediate on the singlet surface and therefore be more likely to behave statistically.

While the quantitative success of the NA-TST implementation in three of the four systems here is likely fortuitous, the qualitative success is likely not. The dominant product channels are rate-limited by a transition state resulting from strong coupling between surfaces of differing multiplicity and the kinetics are similar to those of many purely adiabatic ion–molecule reactions. The increasing reaction efficiency and increasingly negative temperature dependence from X = F to Cl to Br correspond to the increasingly submerged location of the modeled TS located at an ISC. That the MECP geometries used here serve as reasonable proxies for the actual adiabatic transition states suggests that in these systems the adiabatic transition state geometry is not far from that of the MECP. The same cannot be assumed for all reactions, particularly in cases where, unlike here, the slopes of the diabats are of common sign. A more robust implication is that the primary challenge in accurately predicting kinetics from computed potential surfaces for strongly coupled systems is accurate calculation of the relative energy of the rate-limiting transition state. Here, the TST approach is very successful, likely because the calculated MECP energies happen through cancellation of errors to be within chemical accuracy of the true transition states. Similar success should not be expected when repeating this low-calculation cost method for an arbitrary system.

For all of X = F, Cl, Br the determined energy of the rate-limiting transition state lies about 0.15 eV above the calculated energy of the ^3^I^+^(H_3_CX) entrance complex (that is the triplet structure with the iodine atom approaching a hydrogen atom). The constant offset arises because the ^1^CH_2_X^+^(HI) exit complex (i.e., the singlet structure on the “other side” of the transition state) is, for all X, calculated at similar values of about −1.7 eV below reactants (the consistency arising from the similar H–CH_2_X bond energies). The energies of the rate-limiting feature of the reactions are effectively set by the strength of the electrostatic interaction in ^3^I^+^(H_3_CX) (on a triplet surface, no covalent bonding can occur between I^+^ and CH_3_X). That interaction is dominated by the CH_3_X dipole moments, which vary little, and the CH_3_X polarizabilities, which vary significantly. Indeed, the calculated binding energies of ^3^I^+^(CH_3_X) (X = F, Cl, Br) for which the dipole moments are similar, are perfectly correlated to the isotropic polarizabilities. The increasing total rate constants across I^+^ + CH_3_X (X = F, Cl, Br, I) are not due to increased ISC efficiency from increased SOC, but instead due to the deepening entrance well “pulling down” the adiabatic transition state.

The reactions of other halenium cations with the methyl halides, where studied, show less rich chemistry than that of I^+^ reported here, owing to the larger recombination energies of the cations. Cl^+^ reacts with CH_3_Cl exclusively via charge transfer despite the spin-forbidden hydride abstraction channel lying about 2.7 eV lower in energy.^18^ Br^+^ + CH_3_Cl proceeds primarily by charge transfer, but also with a smaller contribution of the energetically preferred, spin-forbidden hydride abstraction channel.^19^ A bond-additivity estimate shows that the CH_2_X^+^(HY) minima, and correspondingly the ISC to hydride abstraction, will be increasingly submerged relative to Y^+^ + CH_3_X for Y = Cl up to F compared to the Y = I systems here. It follows that unlike the Y = I systems, the ISC should not be at too high an energy to ever be rate limiting. However, in these other systems the diabatic transition probability should be increased, both due to larger SOC and increased kinetic energy at the crossing point.

The reactions of transition metal and lanthanide cations with the methyl halides most commonly occur by halogen abstraction.^24,25,62–65^ These are formally spin-allowed reactions, but generally must proceed through a nonspin conserving pathway at low energy (i.e., two-state reactivity). The low-energy kinetics of both the transition metal ion and halenium ion reactions with the methyl halides can be generalized by an interplay of two factors: the energy of the adiabatic transition state and the strength of the spin–orbit coupling. Nonadiabatic effects will be unimportant where the transition state is close to the energy of reactants and the coupling is large (as is the case in the present study) and will become increasingly important as both quantities decrease.

## Conclusions

The kinetics of I^+^ + CH_3_X (X = F, Cl, Br, I) were measured across the temperature range 300–600 K and largely explained through a combination of quantum chemical calculations and statistical modeling. The dominant product channels in the X = F, Cl, and Br reactions are formally spin-forbidden, requiring ISC from triplet to singlet surfaces. The observed kinetics are quantitatively modeled through NA-TST treating calculated MECP as transition states along the adiabatic paths. Calculated spin–orbit couplings at these points are sufficiently large that adiabatic behavior occurs with near unit efficiency and the reactivity at the studied energies is indistinguishable from a spin-conserved process. The X = I reaction occurs primarily via charge transfer (a spin-conserving process).

Measured reaction efficiencies increase starkly from X = F to I. Although these efficiencies correlate with reactant mass and the one predominantly spin-conserving reaction (X = I) is the only one to occur with unit efficiency, none of the reactivity is limited by ISC. Instead, the observed reactivity is in all cases reasonably explained through the calculated energetics, either of products or transition states. At thermal energies, these reactions behave as one would expect an ion–molecule reaction to, given the ground state adiabatic surfaces, without added complication from the varying total spin along those surfaces. Here, the operative transition states are successfully aped by calculating the minimum energy crossing points at a computationally inexpensive level of theory. However, there cannot be confidence that the same approach will succeed in other systems. Instead, confident prediction requires accurate calculation of the adiabatic transition states, a challenging task even employing more computationally intensive methods.

Although not explored in the present experiment, the behavior of these reactions at elevated collision energies will be informative. Extrapolating from the present results, charge transfer may dominate once energetically accessible. However, elevated total energy increases the likelihood of nonadiabatic transitions and may inhibit the “spin-forbidden” channels. Another open question is whether the spin-allowed, but energetically dispreferred methyl abstraction (X + CH_3_I^+^) channel in X = F, Cl, will compete effectively with the formally spin-forbidden channels that dominate at low energy. In X = Cl, this channel opens only about 0.1 eV above separated reactants, while the X = F the channel requires about 1.5 eV.

The observation here of CH_2_I^+^ product from the I^+^ + CH_3_F is attributed to contamination from excited state singlet I^+^. CH_2_I^+^ could be produced in its ground state through a spin-allowed reaction or in an excited triplet state through a formally spin-forbidden reaction, with the two geometries predicted to have significantly different vibrational modes such that identification may be possible. This is a test-reaction to investigate the open question of whether excited state reactants in ion–molecule reactions are commonly trapped on a high-energy adiabat, similar to the behavior previously described for the ^4^Fe^+^ + CH_3_Cl reaction.^25^

## Supplementary Material



## Notes

Quantum chemical calculation results (geometries, energies, frequencies, and comparison to literature) Calculated spin–orbit couplings (PDF)
