# Supplementary material for: Kinetics of Submerged Intersystem Crossings in Strongly Coupled Ion–Molecule Reactions: I+ + CH3X (X = F, Cl, Br, I)
Source: J Phys Chem A. 2026 Jun 9;130(24):4517–27. doi: 10.1021/acs.jpca.6c00949 (PMC13288672; doi:10.1021/acs.jpca.6c00949)
Supplement: Supplementary file 1 [file jp6c00949_si_001.pdf]

Supporting Information for:

Kinetics of submerged intersystem crossings in strongly coupled ion-molecule reactions: I^+ + CH_3X (X = F, Cl, Br, I)

Tucker W. R. Lewis, Albert A. Viggiano, Shaun G. Ard,* and Nicholas S. Shuman*

Air Force Research Laboratory, Space Vehicles Directorate, Kirtland Air Force Base, NM USA 87107

Table S1. Comparison of reaction thermicities calculated at B3LYP/x2c-TZVPPall to experimental values where known for I^+ + CH_3X reactions.

		$\Delta_rH^o_{0K}$ (eV)		
Reactants	Products	Exp.	Calc.	Diff.
$I^+ + CH_3F$	$CH_3F^+ + I$	2.08	1.60	0.48
	$CH_3I^+ + F$	1.39	1.11	0.28
$I^+ + CH_3Cl$	$CH_3Cl^+ + I$	0.84	0.38	0.46
	$CH_3^+ + ICl$	0.80	0.41	0.39
	$Cl + CH_3I^+$	0.24	-0.11	0.36
	$CH_2Cl^+ + HI$	-0.52	-0.99	0.47
$I^+ + CH_3Br$	$CH_3Br^+ + I$	0.09	-0.19	0.28
	$CH_3^+ + IBr$	0.56	0.17	0.39
	$IBr^+ + CH_3$	0.51	0.11	0.40
	$CH_3I^+ + Br$	-0.34	-0.58	0.24
$I^+ + CH_3I$	$CH_3I^+ + I$	-0.91	-1.02	0.11
	$CH_3^+ + I_2$	0.26	-0.12	0.37
	$I_2^+ + CH_3$	-0.28	-0.60	0.33

Table S2. As Table II but including all observed ions, including those that must arise from excited state reactants or contaminants, indicated by '*'.

Reaction	Observed Product	Reaction Type	Branching fraction	Thermicity (eV)
$I^+ + CH_3F$	CH_2F^+	HA	0.75 (+0.12 / -0.18)	-0.5
	*CH_3F^+	CT	0.14 (+0.16 / -0.09)	1.6
	CH_2I^+	CA	0.10 (+0.09 / -0.05)	-1.6
	*CH_3I^+	MA	0.01 (+0.02 / -0.00)	1.2
$I^+ + CH_3Cl$	CH_2Cl^+	HA	0.88 (+0.05 / -0.08)	-0.9

	*CH_3Cl^+	CT	0.01 (+0.01 / -0.01)	0.4
	CH_2I^+	CA	0.08 (+0.06 / -0.04)	-0.9
	CH_3I^+	MA	0.01 (+0.01 / -0.01)	-0.1
	$IClCH_3^+$	AS	0.03 (+0.02 / -0.01)	-2.5
I^+ + CH_3Br	CH_2Br^+	HA	0.45 (+0.15 / -0.17)	-1.0
	CH_3Br^+	CT	0.09 (+0.07 / -0.04)	-0.1
	CH_2I^+	CA	0.03 (+0.03 / -0.01)	-1.0
	CH_3I^+	MA	0.40 (+0.18 / -0.14)	-0.5
	$IBrCH_3^+$	AS	0.02 (+0.02 / -0.01)	-2.5
	IBr^+	XA	0.00 (+0.00 / -0.00)	
I^+ + CH_3I	CH_3I^+	CT/MA	0.96 (+0.02 / -0.10)	-1.0 (-0.5)
	I_2^+	XA	0.02 (+0.02 / -0.01)	
	$CH_3I_2^+$	AS	0.00 (+0.01 / -0.00)	-3.0
	CH_3^+		0.01 (+0.10 / -0.01)	

Table S3. Summary of calculations at B3LYP/x2c-TZVPPall.

CH3F Reference E+ZPE (Hartrees): -7254.84848189

Structure: F-1-F-001

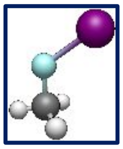

Charge: 1

Multiplicity: 1

Energy (Eh): -7254.92288826

ZPE (Eh): 0.03983022

E + ZPE (Eh): -7254.88305804

Rel. E (eV): -0.9409

SOCME (cm-1): 3496.069222981862

--- GEOMETRY (Angstroms) ---

C 2.538330 -0.335981 -0.000004

H 2.445350 -0.907603 -0.915167

H 3.340625 0.393310 -0.000365

H 2.445667 -0.907055 0.915533

I -0.668685 -0.036652 0.000047

F 1.318212 0.591291 -0.000053

--- FREQUENCIES (cm-1) ---

102.34 174.35 407.49 661.76 1113.66 1124.59 1440.64 1449.62 1454.81 3088.61 3227.75 3237.82

--- ROTATIONAL CONSTANTS (cm-1)---

1.460444 0.088952 0.085279

Structure: F-1-F-002

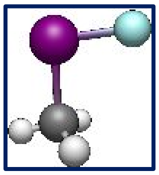

Charge: 1

Multiplicity: 1

Energy (Eh): -7254.94239636

ZPE (Eh): 0.03827497

E + ZPE (Eh): -7254.90412139

Rel. E (eV): -1.5140

SOCME (cm-1): 2855.2407962170964

--- GEOMETRY (Angstroms) ---

C -1.799449 0.616515 0.000023

H -1.846741 1.196690 -0.915573

H -2.496533 -0.222618 -0.000130

H -1.846848 1.196457 0.915761

I 0.131332 -0.329058 -0.000001

F 1.119609 1.267605 0.000062

--- FREQUENCIES (cm-1) ---

97.09 180.35 460.84 662.23 911.34 923.76 1287.32 1419.23 1436.13 3047.76 3164.74 3210.0

--- ROTATIONAL CONSTANTS (cm-1) ---

0.322887 0.210837 0.130898

Structure: F-1-F-003

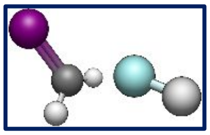

Charge: 1

Multiplicity: 1

Energy (Eh): -7254.95640875

ZPE (Eh): 0.03478269

E + ZPE (Eh): -7254.92162606

Rel. E (eV): -1.9904

SOCME (cm-1): 2003.846356142107

--- GEOMETRY (Angstroms) ---

C -0.849133 1.109403 -0.000313

H -1.262039 1.455960 -0.939563

H -1.258633 1.460512 0.938766

I 0.690126 -0.097689 -0.000018

H -3.715967 -0.540320 0.002737

F -2.792815 -0.431454 -0.000076

--- FREQUENCIES (cm-1) ---

62.11 111.34 151.66 266.4 267.74 735.65 877.89 1006.03 1413.95 3132.76 3271.51 3970.8

--- ROTATIONAL CONSTANTS (cm-1) ---

0.655298 0.071987 0.065762

Structure: F-1-F-TS1

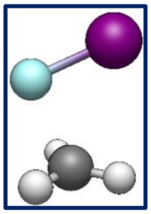

Charge: 1

Multiplicity: 1

Energy (Eh): -7254.87957049

ZPE (Eh): 0.03592391

E + ZPE (Eh): -7254.843647

Rel. E (eV): 0.1315

SOCME (cm-1): 1516.38

--- GEOMETRY (Angstroms) ---

C -1.275655 1.697947 -0.035829

H -0.570291 2.435810 -0.393112

H -1.872937 1.142656 -0.748822

H -1.526436 1.665358 1.016438

I 0.152131 -0.779822 0.791597

F 0.538954 0.529832 -0.630292

--- FREQUENCIES (cm-1) ---

-431.84 13.95 217.86 536.23 576.18 632.45 1287.36 1412.19 1429.06 3090.65 3283.73 3289.08

--- ROTATIONAL CONSTANTS (cm-1) ---

0.491427 0.116884 0.096327

Structure: F-1-F-TS2

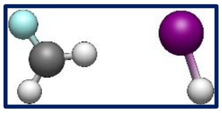

Charge: 1

Multiplicity: 1

Energy (Eh): -7254.91769078

ZPE (Eh): 0.03245873

E + ZPE (Eh): -7254.88523205

Rel. E (eV): -1.0000

SOCME (cm-1): 4.20

--- GEOMETRY (Angstroms) ---

C 2.853606 0.522969 -0.838237

H 3.567234 -0.090785 -1.392052

H 2.416464 -1.163966 3.075186

H 2.518240 0.368922 0.200304

I 1.583402 0.179336 2.713163

F 2.374832 1.495562 -1.451119

--- FREQUENCIES (cm-1) ---

-392.0 41.37 99.48 122.29 246.77 1200.84 1251.59 1405.44 1557.85 2273.99 2881.11 3167.0

--- ROTATIONAL CONSTANTS (cm-1) ---

1.288757 0.037418 0.036362

Structure: F-3-F-001

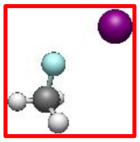

Charge: 1

Multiplicity: 3

Energy (Eh): -7254.92052883

ZPE (Eh): 0.03949915

E + ZPE (Eh): -7254.88102968

Rel. E (eV): -0.8857

SOCME (cm-1): 3660.3612057850246

--- GEOMETRY (Angstroms) ---

C 2.911373 0.292530 -0.000053

H 2.900970 0.892678 -0.903993

H 2.901461 0.891659 0.904568

H 3.721002 -0.433210 -0.000688

I -0.791241 0.036702 0.000047

F 1.706565 -0.510503 -0.000168

--- FREQUENCIES (cm-1) ---

50.5 110.05 199.48 851.05 1148.48 1161.93 1455.63 1456.18 1475.06 3065.69 3171.42 3192.65

--- ROTATIONAL CONSTANTS (cm-1) ---

1.787583 0.063396 0.061967

Structure: F-3-F-002

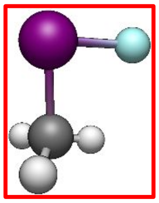

Charge: 1

Multiplicity: 3

Energy (Eh): -7254.87449358

ZPE (Eh): 0.03709841

E + ZPE (Eh): -7254.83739517

Rel. E (eV): 0.3017

SOCME (cm-1): 2892.7846255813793

--- GEOMETRY (Angstroms) ---

C -1.689930 0.803469 -0.000067

H -1.331059 1.827808 0.008591

H -2.220004 0.518901 -0.907584

H -2.228831 0.505819 0.897905

I 0.101426 -0.390856 -0.000744

F 1.170609 1.439615 0.001082

--- FREQUENCIES (cm-1) ---

43.47 153.1 387.34 449.91 859.11 906.81 1275.34 1411.68 1412.52 3038.11 3152.41 3194.54

--- ROTATIONAL CONSTANTS (cm-1) ---

0.254055 0.211634 0.118115

Structure: F-3-F-TS1

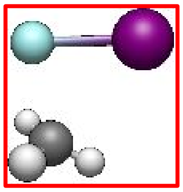

Charge: 1

Multiplicity: 3

Energy (Eh): -7254.83744072

ZPE (Eh): 0.03563933

E + ZPE (Eh): -7254.80180139

Rel. E (eV): 1.2702

SOCME (cm-1): 2970.058861369586

--- GEOMETRY (Angstroms) ---

C 2.241557 -0.751410 -0.000954

H 2.567142 -0.322984 -0.938013

H 2.588798 -0.304537 0.919291

H 1.832117 -1.751048 0.013829

I -0.567504 -0.094853 -0.021975

F 1.141581 1.247993 0.027867

--- FREQUENCIES (cm-1) ---

-379.97 76.85 190.99 332.57 614.28 648.45 1233.4 1420.95 1425.42 3105.38 3297.11 3298.45

--- ROTATIONAL CONSTANTS (cm-1) ---

0.403442 0.117364 0.092654

Structure: F-F-SIDE_MECP

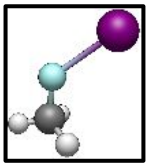

Charge: 1

Multiplicity:

Energy (Eh): -7254.91564286

ZPE (Eh): 0.03975027

E + ZPE (Eh): -7254.87589259

Rel. E (eV): -0.7459

SOCME (cm-1): NaN

--- GEOMETRY (Angstroms) ---

C 2.878609 0.300373 -0.000051

H 2.836168 0.889323 -0.908741

H 2.836664 0.888305 0.909318

H 3.681719 -0.430347 -0.000684

I -0.550756 0.075865 0.000052

F 1.667726 -0.553663 -0.000180

--- FREQUENCIES (cm-1) ---

56.07 126.75 325.35 772.43 1145.55 1146.9 1448.6 1465.82 1466.53 3079.51 3198.73 3216.12

--- ROTATIONAL CONSTANTS (cm-1) ---

1.592341 0.075733 0.073343

Structure: F-3-H-001

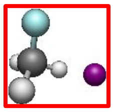

Charge: 1

Multiplicity: 3

Energy (Eh): -7254.90652976

ZPE (Eh): 0.03691753

E + ZPE (Eh): -7254.86961223

Rel. E (eV): -0.5750

SOCME (cm-1): 3360.024188067699

--- GEOMETRY (Angstroms) ---

C 2.464842 0.609074 -0.000146

H 2.675173 1.145093 -0.927653

H 1.297023 0.478045 0.002479

H 2.678468 1.146819 0.925652

I -0.907115 -0.008093 0.000169

F 3.001004 -0.614814 0.000040

--- FREQUENCIES (cm-1) ---

75.46 203.45 244.5 1060.15 1084.31 1152.23 1248.8 1304.8 1471.4 2199.63 3032.98 3127.22

--- ROTATIONAL CONSTANTS (cm-1) ---

1.057227 0.046353 0.044815

Structure: F-3-H-002

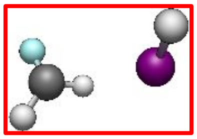

Charge: 1

Multiplicity: 3

Energy (Eh): -7254.85546571

ZPE (Eh): 0.02990483

E + ZPE (Eh): -7254.82556088

Rel. E (eV): 0.6237

SOCME (cm-1): 193.12744496834208

--- GEOMETRY (Angstroms) ---

C 2.415465 0.582819 0.097217

H 2.939077 1.536931 0.014459

H 1.402348 0.538260 -0.406395

I -0.862976 -0.009065 -0.031219

H -0.522908 -0.237049 1.549224

F 3.105533 -0.512750 -0.061543

--- FREQUENCIES (cm-1) ---

39.74 64.95 149.06 232.15 323.3 776.02 1073.31 1255.22 1334.95 2224.5 2568.99 3084.52

--- ROTATIONAL CONSTANTS (cm-1) ---

1.163751 0.046857 0.045723

Structure: F-3-H-TS1

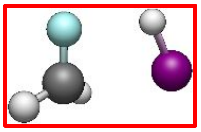

Charge: 1

Multiplicity: 3

Energy (Eh): -7254.85385043

ZPE (Eh): 0.03021169

E + ZPE (Eh): -7254.82363874

Rel. E (eV): 0.6760

SOCME (cm-1): 72.85189359241117

--- GEOMETRY (Angstroms) ---

C 2.414431 0.809854 -0.083638

H 1.641832 0.881204 -0.869425

H 0.224770 -0.739864 0.975710

H 3.325283 1.393486 -0.166045

I -0.829097 0.357709 0.366428

F 2.537764 -0.412283 0.430223

--- FREQUENCIES (cm-1) ---

-183.38 84.48 137.56 152.09 251.69 728.26 1116.83 1171.25 1399.61 2175.19 2872.43 3172.02

--- ROTATIONAL CONSTANTS (cm-1) ---

0.933927 0.057493 0.054375

Structure: F-1-H-001

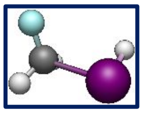

Charge: 1

Multiplicity: 1

Energy (Eh): -7254.96318652

ZPE (Eh): 0.03623602

E + ZPE (Eh): -7254.92695050

Rel. E (eV): -2.1352

SOCME (cm-1): 2214.612607206958

--- GEOMETRY (Angstroms) ---

C -1.603058 0.580488 0.122680

H -1.662510 0.936164 1.147204

H -1.657077 1.326903 -0.665714

I 0.626857 -0.018985 -0.035087

H 0.635215 -0.771841 1.410593

F -2.335150 -0.482655 -0.109248

--- FREQUENCIES (cm-1) ---

133.94 211.41 417.35 535.45 897.91 1176.85 1233.5 1273.94 1492.73 2233.97 3091.37 3207.34

--- ROTATIONAL CONSTANTS (cm-1) ---

1.061943 0.087130 0.083850

Structure: F-H-SIDE_MECP

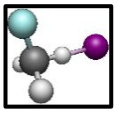

Charge: 1

Multiplicity:

Energy (Eh): -7254.90336341

ZPE (Eh): 0.03626190

E + ZPE (Eh): -7254.86710151

Rel. E (eV): -0.5067

SOCME (cm-1): 3207.5785331617367

--- GEOMETRY (Angstroms) ---

C 2.462699 0.594997 -0.000276

H 2.613602 1.134244 -0.937279

H 1.238350 0.431476 0.001430

H 2.615597 1.135769 0.935546

I -0.730560 0.064262 0.000997

F 3.009707 -0.604624 0.000122

--- FREQUENCIES (cm-1) ---

49.22 282.13 299.72 1035.99 1089.42 1183.33 1238.68 1250.49 1478.53 1835.56 3031.44 3142.64

--- ROTATIONAL CONSTANTS (cm-1) ---

1.112712 0.050989 0.049258

Structure: F-3-TS-Fside_Hside

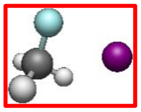

Charge: 1

Multiplicity: 3

Energy (Eh): -7254.90540374

ZPE (Eh): 0.03716834

E + ZPE (Eh): -7254.86823540

Rel. E (eV): -0.5375

SOCME (cm-1): 3480.1388047030537

--- GEOMETRY (Angstroms) ---

C	2.392475	-0.608069	-0.000966
H	2.840809	-0.962534	0.927246
H	1.323278	-1.036141	-0.015969
H	2.863701	-0.968069	-0.915948
I	-0.780382	0.001285	0.011536
F	2.286380	0.748690	-0.006113

--- FREQUENCIES (cm-1) ---

-94.9 179.2 203.47 1007.5 1108.66 1124.36 1244.2 1389.9 1466.95 2399.26 3052.02 3139.51

--- ROTATIONAL CONSTANTS (cm-1) ---

0.870580 0.064074 0.060414

Structure: F-1-TS-Fside_Hside

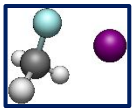

Charge: 1

Multiplicity: 1

Energy (Eh): -7254.87152191

ZPE (Eh): 0.03614692

E + ZPE (Eh): -7254.83537499

Rel. E (eV): 0.3567

SOCME (cm-1): 3520.622217733678

--- GEOMETRY (Angstroms) ---

C -2.104839 0.580676 0.002342

H -2.599375 0.841678 -0.930132

H -2.652123 0.830059 0.908739

H -1.127372 1.183976 0.040328

I 0.657644 0.014730 -0.020164

F -1.758030 -0.791588 0.002660

--- FREQUENCIES (cm-1) ---

-1028.59 14.78 226.43 793.93 1105.06 1119.6 1195.55 1453.57 1494.62 2192.07 3086.33 3184.74

--- ROTATIONAL CONSTANTS (cm-1) ---

0.839355 0.092634 0.084860

Structure: H3CF

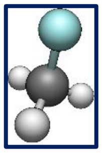

Charge: 0

Multiplicity: 1

Energy (Eh): -139.84488273

ZPE (Eh): 0.03906863

E + ZPE (Eh): -139.80581410

SOCME (cm-1): 48.07775718562587

--- GEOMETRY (Angstroms) ---

C 2.395203 0.614333 0.000112

H 2.702017 1.156780 -0.894494

H 1.313631 0.476934 0.002500

H 2.705747 1.158507 0.892378

F 3.005900 -0.633625 0.000043

--- FREQUENCIES (cm-1) ---

1050.16 1192.07 1192.3 1486.4 1492.23 1492.77 3028.1 3107.5 3107.61

--- ROTATIONAL CONSTANTS (cm-1) ---

5.236162 0.851208 0.851165

Structure: CH3F+

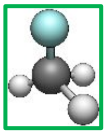

Charge: 1

Multiplicity: 2

Energy (Eh): -139.39175059

ZPE (Eh): 0.03282718

E + ZPE (Eh): -139.35892341

--- GEOMETRY (Angstroms) ---

C 0.566314 -0.083014 0.000080

H 1.037381 0.707231 -0.724022

H 1.036719 0.708333 0.723685

H 0.979281 -1.088189 0.001011

F -0.716648 0.019158 -0.000158

--- FREQUENCIES (cm-1) ---

763.99 961.64 995.38 1069.63 1302.72 1459.36 2156.09 2519.91 3180.75

--- ROTATIONAL CONSTANTS (cm-1) ---

5.058858 0.968554 0.905126

Structure: CH2F+

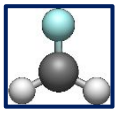

Charge: 1

Multiplicity: 1

Energy (Eh): -138.84607981

ZPE (Eh): 0.02686130

E + ZPE (Eh): -138.81921851

SOCME (cm-1): 78.40000000000006

--- GEOMETRY (Angstroms) ---

C -0.000000 0.000000 -0.593648

F 0.000000 -0.000000 0.637883

H 0.000000 0.974877 -1.092517

H 0.000000 -0.974877 -1.092517

--- FREQUENCIES (cm-1) ---

1242.55 1287.77 1440.35 1595.36 3030.67 3194.05

--- ROTATIONAL CONSTANTS (cm-1) ---

8.798453 1.192669 1.050297

Structure: CH2F+

Charge: 1

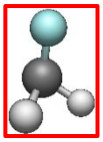

Multiplicity: 3

Energy (Eh): -138.69989903

ZPE (Eh): 0.01930637

E + ZPE (Eh): -138.68059266

SOCME (cm-1): 63.17475761726361

--- GEOMETRY (Angstroms) ---

C -0.081505 0.601469 0.000000

F -0.081271 -0.650184 -0.000000

H 0.610471 1.122256 0.806411

H 0.610471 1.122255 -0.806411

--- FREQUENCIES (cm-1) ---

777.83 890.51 997.69 1436.96 1996.57 2374.96

--- ROTATIONAL CONSTANTS (cm-1) ---

8.370591 1.082505 1.041823

Structure: HF

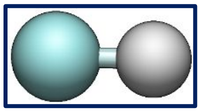

Charge: 0

Multiplicity: 1

Energy (Eh): -100.54340230

ZPE (Eh): 0.00929115

E + ZPE (Eh): -100.53411115

SOCME (cm-1): 75.84235294345767

--- GEOMETRY (Angstroms) ---

F 0.000000 0.000000 0.091368

H 0.000000 0.000000 -0.830996

--- FREQUENCIES (cm-1) ---

4078.34

--- ROTATIONAL CONSTANTS (cm-1) ---

0.000000 20.700614 20.700614

Structure: F

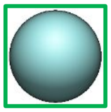

Charge: 0

Multiplicity: 2

Energy (Eh): -99.82495548

ZPE (Eh): 0.00000000

E + ZPE (Eh): -99.82495548

SOCME (cm-1): 222.91

CH3Cl Reference E+ZPE (Hartrees): -7616.50175956

Structure: Cl-1-Cl-001

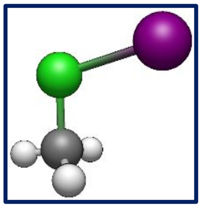

Charge: 1

Multiplicity: 1

Energy (Eh): -7616.61547972

ZPE (Eh): 0.03862229

E + ZPE (Eh): -7616.57685743

Rel. E (eV): -2.0435

SOCME (cm-1): 3009.6073673356464

--- GEOMETRY (Angstroms) ---

C 2.478994 0.816062 0.000059

H 2.252385 1.355820 0.911841

H 3.474871 0.377417 0.001058

H 2.253561 1.354619 -0.912719

Cl 1.398511 -0.688462 0.000377

I -0.881128 0.071039 -0.000415

--- FREQUENCIES (cm-1) ---

141.18 162.11 322.89 549.68 1019.82 1023.95 1361.5 1437.03 1452.35 3068.75 3189.82 3224.14

--- ROTATIONAL CONSTANTS (cm-1) ---

0.553722 0.064125 0.058135

Structure: Cl-1-Cl-002

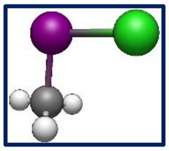

Charge: 1

Multiplicity: 1

Energy (Eh): -7616.62190667

ZPE (Eh): 0.03771058

E + ZPE (Eh): -7616.58419609

Rel. E (eV): -2.2432

SOCME (cm-1): 1989.4974279641579

--- GEOMETRY (Angstroms) ---

C -1.394432 1.492879 -0.000013

H -1.100034 1.997742 -0.912856

H -2.441617 1.194696 -0.000094

H -1.100130 1.997618 0.912927

I -0.337518 -0.401594 -0.000101

Cl 1.819496 0.410439 0.000116

--- FREQUENCIES (cm-1) ---

107.28 147.11 390.52 454.68 914.82 919.11 1285.94 1426.95 1442.45 3061.63 3182.48 3220.06

--- ROTATIONAL CONSTANTS (cm-1) ---

0.271286 0.101216 0.074813

Structure: Cl-1-Cl-003

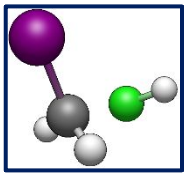

Charge: 1

Multiplicity: 1

Energy (Eh): -7616.60208768

ZPE (Eh): 0.03424365

E + ZPE (Eh): -7616.56784403

Rel. E (eV): -1.7983

SOCME (cm-1): 1580.718520167332

--- GEOMETRY (Angstroms) ---

C -0.718352 1.008829 0.063535

H -0.961374 1.557420 -0.837199

H -0.976171 1.468451 1.007826

I 0.970438 -0.116278 0.012445

Cl -2.593550 -0.180897 -0.068380

H -2.493308 -0.825767 1.050140

--- FREQUENCIES (cm-1) ---

63.26 123.89 223.81 539.71 627.81 765.11 955.57 1114.67 1396.45 2824.97 3135.75 3260.22

--- ROTATIONAL CONSTANTS (cm-1) ---

0.731769 0.045114 0.043148

Structure: Cl-1-Cl-TS1

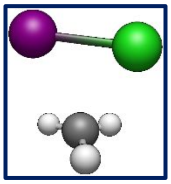

Charge: 1

Multiplicity: 1

Energy (Eh): -7616.56018442

ZPE (Eh): 0.03565693

E + ZPE (Eh): -7616.52452749

Rel. E (eV): -0.6195

SOCME (cm-1): 485.25148119299956

--- GEOMETRY (Angstroms) ---

C -1.294686 1.707159 -0.005632

H -0.629626 2.503483 -0.305340

H -1.881233 1.193971 -0.755781

H -1.577249 1.626347 1.032835

I 0.066605 -0.741609 1.005828

Cl 0.761955 0.402429 -0.971930

--- FREQUENCIES (cm-1) ---

-408.22 40.96 241.2 364.94 565.18 644.78 1235.88 1415.37 1432.1 3108.5 3296.58 3306.1

--- ROTATIONAL CONSTANTS (cm-1) ---

0.223350 0.087831 0.063877

Structure: Cl-1-Cl-TS2

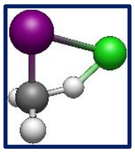

Charge: 1

Multiplicity: 1

Energy (Eh): -7616.52268325

ZPE (Eh): 0.03056679

E + ZPE (Eh): -7616.49211646

Rel. E (eV): 0.2624

SOCME (cm-1): 628.9696018091812

--- GEOMETRY (Angstroms) ---

C -0.545354 1.588129 0.516620

H -0.676932 2.370097 -0.222508

H -0.498946 1.842514 1.569264

I 0.628441 0.047423 -0.050538

Cl -1.976838 -0.849966 -0.041539

H -1.621366 0.644816 0.344067

--- FREQUENCIES (cm-1) ---

-2130.79 139.09 389.62 464.62 654.21 898.97 921.36 991.35 1220.19 1387.53 3108.29 3242.05

--- ROTATIONAL CONSTANTS (cm-1) ---

0.304789 0.078774 0.063431

Structure: Cl-3-Cl-001

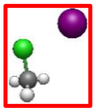

Charge: 1

Multiplicity: 3

Energy (Eh): -7616.59205472

ZPE (Eh): 0.03797698

E + ZPE (Eh): -7616.55407774

Rel. E (eV): -1.4237

SOCME (cm-1): 3277.8194039550135

--- GEOMETRY (Angstroms) ---

C -2.828477 0.829126 0.000007

H -2.613003 1.383834 0.905704

H -2.613752 1.383104 -0.906312

H -3.842251 0.434999 0.000605

Cl -1.776568 -0.658865 0.000158

I 1.062207 0.058253 -0.000129

--- FREQUENCIES (cm-1) ---

65.82 88.03 161.43 612.85 1003.87 1019.73 1363.94 1444.79 1455.51 3069.07 3176.27 3208.64

--- ROTATIONAL CONSTANTS (cm-1) ---

0.575791 0.044681 0.041804

Structure: Cl-3-Cl-002

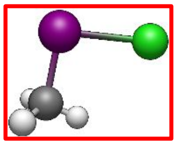

Charge: 1

Multiplicity: 3

Energy (Eh): -7616.56679625

ZPE (Eh): 0.03686464

E + ZPE (Eh): -7616.52993161

Rel. E (eV): -0.7666

SOCME (cm-1): 1319.4049310579371

--- GEOMETRY (Angstroms) ---

C -1.199639 1.666064 0.000136

H -0.308894 2.284814 -0.000940

H -1.791474 1.758535 -0.905284

H -1.790104 1.759732 0.906329

I -0.483789 -0.381721 0.000930

Cl 2.160438 0.252194 -0.001100

--- FREQUENCIES (cm-1) ---

36.68 84.38 207.8 458.35 890.89 902.33 1281.35 1430.46 1430.87 3065.0 3190.98 3202.63

--- ROTATIONAL CONSTANTS (cm-1) ---

0.257966 0.076118 0.059461

Structure: Cl-3-Cl-TS1

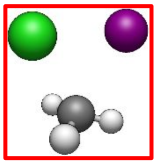

Charge: 1

Multiplicity: 3

Energy (Eh): -7616.51795060

ZPE (Eh): 0.03507414

E + ZPE (Eh): -7616.48287646

Rel. E (eV): 0.5138

SOCME (cm-1): 2110.704147908939

--- GEOMETRY (Angstroms) ---

C 1.236664 1.710798 -0.007025

H 2.005945 1.642400 0.750890

H 1.504401 1.740707 -1.052518

H 0.346032 2.248558 0.309198

I -0.817497 -0.069473 -0.025793

Cl 1.878002 -0.706475 0.024911

--- FREQUENCIES (cm-1) ---

-478.45 79.79 206.01 227.33 638.63 730.98 1156.23 1380.29 1416.52 3064.34 3207.68 3287.97

--- ROTATIONAL CONSTANTS (cm-1) ---

0.257909 0.071723 0.056791

Structure: Cl-Cl-SIDE_MECP

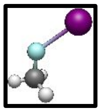

Charge: 1

Multiplicity:

Energy (Eh): -7616.59195942

ZPE (Eh): 0.03802791

E + ZPE (Eh): -7616.55393151

Rel. E (eV): -1.4197

SOCME (cm-1): NaN

--- GEOMETRY (Angstroms) ---

C 2.545975 0.808227 0.000089

H 2.325675 1.360697 0.906005

H 3.561984 0.419892 0.000896

H 2.326645 1.359520 -0.906778

Cl 1.505047 -0.689525 0.000489

I -1.288132 0.027684 -0.000499

--- FREQUENCIES (cm-1) ---

70.37 89.81 173.46 610.69 1005.72 1019.55 1364.43 1447.12 1455.26 3069.51 3177.18 3209.23

--- ROTATIONAL CONSTANTS (cm-1) ---

0.570075 0.046073 0.042988

Structure: Cl-1-H-001

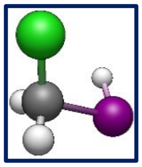

Charge: 1

Multiplicity: 1

Energy (Eh): -7616.61270506

ZPE (Eh): 0.03437009

E + ZPE (Eh): -7616.57833497

Rel. E (eV): -2.0837

SOCME (cm-1): 1135.5175031676085

--- GEOMETRY (Angstroms) ---

C -1.197294 0.868178 0.114172

Cl -2.430045 -0.294417 -0.046793

H -1.141286 1.322462 1.095750

H -1.134935 1.562498 -0.716368

I 0.942416 -0.044273 -0.033589

H 0.763295 -0.909404 1.335135

--- FREQUENCIES (cm-1) ---

121.01 154.62 346.83 523.98 801.84 833.68 1108.77 1186.31 1438.09 2237.92 3114.65 3219.03

--- ROTATIONAL CONSTANTS (cm-1) ---

0.796482 0.048922 0.047066

Structure: Cl-3-H-001

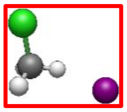

Charge: 1

Multiplicity: 3

Energy (Eh): -7616.56985509

ZPE (Eh): 0.03611384

E + ZPE (Eh): -7616.53374125

Rel. E (eV): -0.8703

SOCME (cm-1): 3227.6326459031857

--- GEOMETRY (Angstroms) ---

C 2.099548 0.890848 0.000005

H 2.167248 1.477253 -0.912347

H 1.087184 0.381813 -0.000258

H 2.166946 1.477004 0.912533

Cl 3.289390 -0.379241 0.000012

I -1.390557 -0.041782 -0.000001

--- FREQUENCIES (cm-1) ---

55.7 119.25 211.85 763.13 934.25 983.83 1267.34 1272.82 1431.37 2559.35 3086.07 3167.18

--- ROTATIONAL CONSTANTS (cm-1) ---

0.808511 0.024446 0.023842

Structure: Cl-3-H-002

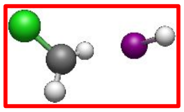

Charge: 1

Multiplicity: 3

Energy (Eh): -7616.52264963

ZPE (Eh): 0.02791957

E + ZPE (Eh): -7616.49473006

Rel. E (eV): 0.1913

SOCME (cm-1): 112.6602414341457

--- GEOMETRY (Angstroms) ---

C	2.085883	0.777107	0.033284
Cl	3.349740	-0.295574	-0.002313
H	2.243259	1.837799	-0.123951
H	1.077467	0.348400	-0.185222
I	-1.342374	-0.039306	-0.030344
H	-1.257591	0.068050	1.593337

--- FREQUENCIES (cm-1) ---

57.5 61.97 121.06 175.95 298.9 408.82 876.91 898.55 1255.87 2239.93 2689.89 3169.92

--- ROTATIONAL CONSTANTS (cm-1) ---

1.012150 0.024576 0.024178

Structure: Cl-3-H-TS1

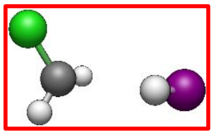

Charge: 1

Multiplicity: 3

Energy (Eh): -7616.51891822

ZPE (Eh): 0.02871798

E + ZPE (Eh): -7616.49020024

Rel. E (eV): 0.3145

SOCME (cm-1): 600.2624426032334

--- GEOMETRY (Angstroms) ---

C 2.284677 0.744217 0.593467

H 1.620245 1.065779 -0.215135

H -0.020909 -0.452482 0.881431

H 2.409158 1.370503 1.469348

Cl 3.582230 -0.209378 0.124340

I -1.256419 -0.036233 -0.108502

--- FREQUENCIES (cm-1) ---

-287.3 31.51 72.21 161.45 291.08 631.3 848.13 933.01 1330.87 2138.24 2985.54 3182.41

--- ROTATIONAL CONSTANTS (cm-1) ---

0.990486 0.023024 0.022612

Structure: Cl-H-SIDE_MECP

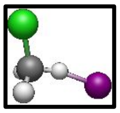

Charge: 1

Multiplicity:

Energy (Eh): -7616.56333447

ZPE (Eh): 0.03490666

E + ZPE (Eh): -7616.52842781

Rel. E (eV): -0.7257

SOCME (cm-1): NaN

--- GEOMETRY (Angstroms) ---

C 2.083720 0.882965 0.000024

H 2.084736 1.458543 -0.922414

H 0.978523 0.373861 -0.000212

H 2.084512 1.458323 0.922598

Cl 3.207239 -0.396305 0.000001

I -1.018969 0.028508 -0.000053

--- FREQUENCIES (cm-1) ---

52.07 242.25 292.89 837.99 963.53 1002.39 1168.96 1203.64 1425.19 1888.08 3071.98 3173.28

--- ROTATIONAL CONSTANTS (cm-1) ---

0.828975 0.030124 0.029241

Structure: Cl-3-TS-Clside_Hside

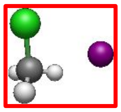

Charge: 1

Multiplicity: 3

Energy (Eh): -7616.56619009

ZPE (Eh): 0.03542559

E + ZPE (Eh): -7616.53076450

Rel. E (eV): -0.7893

SOCME (cm-1): 3216.880476486498

--- GEOMETRY (Angstroms) ---

C 2.213659 0.969853 0.003292

H 2.385393 1.684694 -0.797139

H 1.251566 0.429457 -0.236610

H 2.092507 1.413307 0.988104

Cl 3.488715 -0.234419 0.029369

I 0.002514 -1.587086 -0.747532

--- FREQUENCIES (cm-1) ---

-130.4 116.96 124.91 696.48 905.13 968.06 1234.7 1308.54 1424.89 2519.03 3085.08 3166.26

--- ROTATIONAL CONSTANTS (cm-1) ---

0.497175 0.035142 0.033039

Structure: Cl-1-TS-Clside_Hside

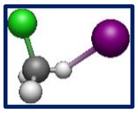

Charge: 1

Multiplicity: 1

Energy (Eh): -7616.53229015

ZPE (Eh): 0.03407630

E + ZPE (Eh): -7616.49821385

Rel. E (eV): 0.0965

SOCME (cm-1): 3068.305721403915

--- GEOMETRY (Angstroms) ---

C 2.006818 0.268903 0.401441

H 1.904893 0.766254 1.357906

H 2.980825 0.409768 -0.072247

H 1.324432 0.788172 -0.374494

Cl 1.601529 -1.484752 0.537472

I -0.684104 0.368915 -0.194208

--- FREQUENCIES (cm-1) ---

-1218.86 148.09 257.22 573.56 862.83 961.78 1180.56 1353.19 1435.62 1938.36 3050.98 3195.56

--- ROTATIONAL CONSTANTS (cm-1) ---

0.463620 0.057123 0.051399

Structure: ICl

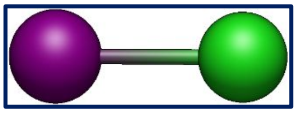

Charge: 0

Multiplicity: 1

Energy (Eh): -7577.03646846

ZPE (Eh): 0.00085295

E + ZPE (Eh): -7577.03561551

SOCME (cm-1): 2333.7394884605264

--- GEOMETRY (Angstroms) ---

I -0.888258 -0.512836 -1.465013

Cl -0.004394 -0.002537 0.650333

--- FREQUENCIES (cm-1) ---

374.4

--- ROTATIONAL CONSTANTS (cm-1) ---

0.000000 0.110279 0.110279

Structure: CH3Cl

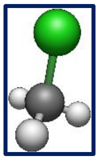

Charge: 0

Multiplicity: 1

Energy (Eh): -501.49667800

ZPE (Eh): 0.03758623

E + ZPE (Eh): -501.45909177

SOCME (cm-1): 239.0179593670737

--- GEOMETRY (Angstroms) ---

C -0.000000 0.000007 -1.134273

H 0.000000 1.030026 -1.475975

H 0.892027 -0.515013 -1.475963

H -0.892027 -0.515013 -1.475963

Cl 0.000000 -0.000007 0.661800

--- FREQUENCIES (cm-1) ---

710.72 1023.68 1023.84 1378.14 1482.82 1482.91 3068.76 3163.65 3163.93

--- ROTATIONAL CONSTANTS (cm-1) ---

5.254357 0.436724 0.436724

Structure: CH3Cl+

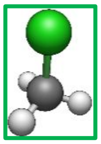

Charge: 1

Multiplicity: 2

Energy (Eh): -501.09006864

ZPE (Eh): 0.03285212

E + ZPE (Eh): -501.05721652

SOCME (cm-1): NaN

--- GEOMETRY (Angstroms) ---

C -0.000000 -0.057865 -1.130631

H 0.000001 1.022243 -1.428739

H 0.935425 -0.498341 -1.476020

H -0.935425 -0.498342 -1.476019

Cl -0.000000 0.032304 0.611037

--- FREQUENCIES (cm-1) ---

300.21 643.93 810.49 1149.97 1221.32 1413.84 2767.42 3047.57 3065.68

--- ROTATIONAL CONSTANTS (cm-1) ---

5.067779 0.463109 0.460558

Structure: CH2Cl+

Charge: 1

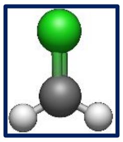

Multiplicity: 1

Energy (Eh): -500.51356878

ZPE (Eh): 0.02509132

E + ZPE (Eh): -500.48847746

SOCME (cm-1): 237.86996447639203

--- GEOMETRY (Angstroms) ---

C 0.248484 0.143475 -0.985375

H -0.122547 1.026746 -1.502950

H 0.822863 -0.620975 -1.506346

Cl -0.056148 -0.033874 0.569826

--- FREQUENCIES (cm-1) ---

1042.74 1046.11 1153.23 1474.57 3080.84 3216.33

--- ROTATIONAL CONSTANTS (cm-1) ---

9.268388 0.590280 0.554937

Structure: CH2Cl+

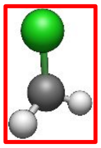

Charge: 1

Multiplicity: 3

Energy (Eh): -500.40694468

ZPE (Eh): 0.02039184

E + ZPE (Eh): -500.38655284

SOCME (cm-1): 241.1135831926522

--- GEOMETRY (Angstroms) ---

C 0.094005 0.053918 -1.046187

H -0.060235 1.053006 -1.492905

H 0.881758 -0.578761 -1.493866

Cl -0.022876 -0.012790 0.608113

--- FREQUENCIES (cm-1) ---

538.34 662.13 764.81 1144.62 2882.23 2958.85

--- ROTATIONAL CONSTANTS (cm-1) ---

8.607063 0.550678 0.523003

Structure: HCl

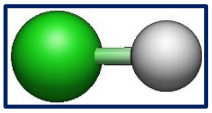

Charge: 0

Multiplicity: 1

Energy (Eh): -462.18808051

ZPE (Eh): 0.00669776

E + ZPE (Eh): -462.18138275

SOCME (cm-1): 236.22023622035434

--- GEOMETRY (Angstroms) ---

H -0.687365 -0.396851 -0.984218

Cl -0.205287 -0.118522 0.169538

--- FREQUENCIES (cm-1) ---

2939.98

--- ROTATIONAL CONSTANTS (cm-1) ---

0.000000 10.480901 10.480901

Structure: Cl

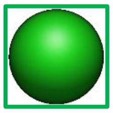

Charge: 0

Multiplicity: 2

Energy (Eh): -461.52306837

ZPE (Eh): 0.00000000

E + ZPE (Eh): -461.52306837

SOCME (cm-1): NaN

--- GEOMETRY (Angstroms) ---

Cl 0.000000 0.000000 0.000000

CH3Br Reference E+ZPE (Hartrees): -9761.07158382

Structure: Br-1-Br-001

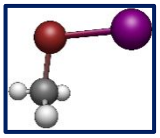

Charge: 1

Multiplicity: 1

Energy (Eh): -9761.19879015

ZPE (Eh): 0.03785316

E + ZPE (Eh): -9761.16093699

Rel. E (eV): -2.4314

SOCME (cm-1): 2500.1027823871564

--- GEOMETRY (Angstroms) ---

C 2.163416 1.302369 -0.000255

H 1.848885 1.791594 0.913241

H 3.214584 1.023864 -0.000721

H 1.848581 1.793324 -0.912719

Br 1.260961 -0.486577 -0.001801

I -1.205004 0.089133 0.001927

--- FREQUENCIES (cm-1) ---

122.47 136.46 238.12 480.0 965.87 967.73 1320.99 1433.95 1447.67 3072.51 3198.05 3231.79

--- ROTATIONAL CONSTANTS (cm-1) ---

0.350103 0.043676 0.039135

Structure: Br-1-Br-002

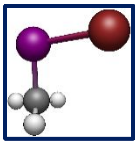

Charge: 1

Multiplicity: 1

Energy (Eh): -9761.20424648

ZPE (Eh): 0.03740273

E + ZPE (Eh): -9761.16684375

Rel. E (eV): -2.5922

SOCME (cm-1): 702.8207453958371

--- GEOMETRY (Angstroms) ---

C 1.605398 1.623916 0.000003

H 1.251206 2.089415 0.912060

H 2.680600 1.456045 -0.000046

H 1.251127 2.089414 -0.912024

I 0.789354 -0.390944 0.000036

Br -1.619359 0.150398 -0.000038

--- FREQUENCIES (cm-1) ---

103.51 129.39 265.02 454.58 913.4 917.17 1284.77 1429.62 1444.02 3065.93 3187.29 3223.2

--- ROTATIONAL CONSTANTS (cm-1) ---

0.265312 0.050657 0.042898

Structure: Br-1-Br-003

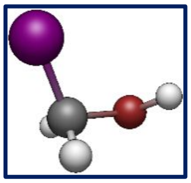

Charge: 1

Multiplicity: 1

Energy (Eh): -9761.17480154

ZPE (Eh): 0.03380975

E + ZPE (Eh): -9761.14099179

Rel. E (eV): -1.8887

SOCME (cm-1): 1528.548625363289

--- GEOMETRY (Angstroms) ---

C -0.236993 1.047123 0.088674

H -0.424282 1.653698 -0.788919

H -0.417112 1.531850 1.038695

I 1.474041 -0.095388 -0.002997

H -1.870028 -0.923875 1.116289

Br -2.124341 -0.101459 -0.034746

--- FREQUENCIES (cm-1) ---

93.63 126.61 269.44 541.38 606.22 771.71 1013.81 1143.14 1395.1 2507.88 3128.48 3243.36

--- ROTATIONAL CONSTANTS (cm-1) ---

0.698520 0.026201 0.025493

Structure: Br-1-Br-TS1

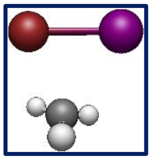

Charge: 1

Multiplicity: 1

Energy (Eh): -9761.14369850

ZPE (Eh): 0.03535573

E + ZPE (Eh): -9761.10834277

Rel. E (eV): -1.0003

SOCME (cm-1): 63.389194662813

--- GEOMETRY (Angstroms) ---

C 2.080476 1.498179 -0.040092

H 1.962773 1.721284 1.011299

H 2.906854 0.884018 -0.364210

H 1.583499 2.117038 -0.770049

Br 1.236001 -1.053287 0.576147

I -0.638177 0.346475 -0.413421

--- FREQUENCIES (cm-1) ---

-375.37 41.18 237.51 257.48 557.97 641.09 1210.63 1413.02 1435.28 3113.99 3300.7 3310.52

--- ROTATIONAL CONSTANTS (cm-1) ---

0.180146 0.051802 0.040572

Structure: Br-1-Br-TS2

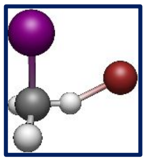

Charge: 1

Multiplicity: 1

Energy (Eh): -9761.10635248

ZPE (Eh): 0.03089687

E + ZPE (Eh): -9761.07545561

Rel. E (eV): -0.1054

SOCME (cm-1): 1370.0835158485777

--- GEOMETRY (Angstroms) ---

C 0.047648 1.641225 -0.000289

H 0.173058 2.189354 -0.928944

H 0.166077 2.184380 0.932020

I 0.949698 -0.196134 -0.000650

Br -2.340937 -0.187707 0.005788

H -1.109023 1.211644 -0.007623

--- FREQUENCIES (cm-1) ---

-1304.2 91.89 355.85 430.44 675.86 897.56 1094.65 1123.53 1193.61 1390.91 3094.98 3212.87

--- ROTATIONAL CONSTANTS (cm-1) ---

0.325130 0.031485 0.028877

Structure: Br-3-Br-001

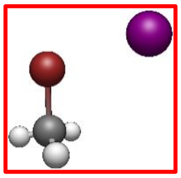

Charge: 1

Multiplicity: 3

Energy (Eh): -9761.17168087

ZPE (Eh): 0.03731480

E + ZPE (Eh): -9761.13436607

Rel. E (eV): -1.7084

SOCME (cm-1): 2947.255402913022

--- GEOMETRY (Angstroms) ---

C 3.320446 0.158900 0.003531

H 3.223214 0.798983 0.871739

H 4.179324 -0.502678 0.070678

H 3.277849 0.689301 -0.939664

Br 1.774463 -1.082047 0.030808

I -0.737345 0.628776 -0.033390

--- FREQUENCIES (cm-1) ---

51.39 73.4 118.37 519.04 948.42 961.47 1321.67 1443.29 1449.83 3077.89 3193.85 3220.67

--- ROTATIONAL CONSTANTS (cm-1) ---

0.363752 0.030629 0.028409

Structure: Br-3-Br-002

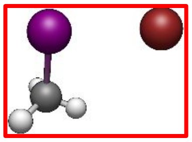

Charge: 1

Multiplicity: 3

Energy (Eh): -9761.15929788

ZPE (Eh): 0.03668415

E + ZPE (Eh): -9761.12261373

Rel. E (eV): -1.3886

SOCME (cm-1): 197.12256198824628

--- GEOMETRY (Angstroms) ---

C -1.653057 1.686209 0.000002

H -0.757377 2.297283 -0.000587

H -2.241038 1.792943 -0.905300

H -2.240094 1.793279 0.905874

I -0.971165 -0.374769 0.000003

Br 1.909084 0.105763 -0.000003

--- FREQUENCIES (cm-1) ---

-24.5 70.13 136.75 465.51 897.41 898.61 1282.68 1436.75 1437.71 3072.72 3200.08 3204.13

--- ROTATIONAL CONSTANTS (cm-1) ---

0.260853 0.036893 0.032528

Structure: Br-3-Br-TS1

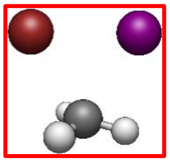

Charge: 1

Multiplicity: 3

Energy (Eh): -9761.10562991

ZPE (Eh): 0.03477339

E + ZPE (Eh): -9761.07085652

Rel. E (eV): 0.0198

SOCME (cm-1): 1291.9978482954218

--- GEOMETRY (Angstroms) ---

C -0.494995 2.036481 0.008977

H -1.326895 2.127728 -0.676980

H -0.659045 2.124405 1.072364

H 0.459823 2.351079 -0.398448

I 1.214731 -0.154476 -0.009523

Br -1.706132 -0.313491 0.006672

--- FREQUENCIES (cm-1) ---

-472.15 76.56 155.28 207.95 592.48 703.45 1133.11 1383.66 1421.48 3079.0 3221.53 3289.26

--- ROTATIONAL CONSTANTS (cm-1) ---

0.220992 0.039520 0.033764

Structure: Br-Br-Side_MECP

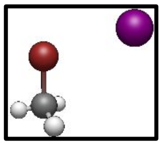

Charge: 1

Multiplicity:

Energy (Eh): -9761.17088730

ZPE (Eh): 0.03718389

E + ZPE (Eh): -9761.13370341

Rel. E (eV): -1.6904

SOCME (cm-1): 3021.9613250337934

--- GEOMETRY (Angstroms) ---

C 3.345603 0.150286 0.001383

H 3.243512 0.798929 0.862726

H 4.204140 -0.510105 0.084189

H 3.319015 0.674672 -0.945743

Br 1.797054 -1.080722 0.020446

I -0.871373 0.658176 -0.019300

--- FREQUENCIES (cm-1) ---

32.4 67.89 94.1 523.64 942.69 962.7 1321.63 1439.38 1450.47 3076.85 3191.04 3219.05

--- ROTATIONAL CONSTANTS (cm-1) ---

0.370726 0.027893 0.026075

Structure: Br-1-H-001

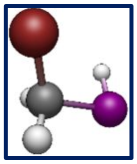

Charge: 1

Multiplicity: 1

Energy (Eh): -9761.18104440

ZPE (Eh): 0.03373441

E + ZPE (Eh): -9761.14730999

Rel. E (eV): -2.0606

SOCME (cm-1): 1060.685575465227

--- GEOMETRY (Angstroms) ---

C -0.604453 1.003831 0.111669

H -0.518149 1.481369 1.079055

H -0.511260 1.675136 -0.734026

I 1.435898 -0.059771 -0.032145

H 1.171084 -0.949262 1.306736

Br -2.076241 -0.146071 -0.021217

--- FREQUENCIES (cm-1) ---

112.35 134.81 338.53 530.3 657.76 799.54 1070.73 1166.48 1412.03 2237.42 3120.6 3227.15

--- ROTATIONAL CONSTANTS (cm-1) ---

0.708492 0.027380 0.026667

Structure: Br-3-H-001

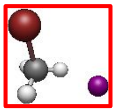

Charge: 1

Multiplicity: 3

Energy (Eh): -9761.14739426

ZPE (Eh): 0.03609997

E + ZPE (Eh): -9761.11129429

Rel. E (eV): -1.0806

SOCME (cm-1): 3164.3560238689956

--- GEOMETRY (Angstroms) ---

C 1.492594 1.040856 0.000104

H 1.536433 1.628667 -0.910239

H 0.575000 0.414708 0.000010

H 1.536365 1.628501 0.910554

I -2.186184 -0.054118 -0.000048

Br 2.957001 -0.202513 0.000048

--- FREQUENCIES (cm-1) ---

28.76 79.07 166.43 607.08 889.66 934.67 1279.58 1315.33 1427.79 2810.95 3115.75 3190.98

--- ROTATIONAL CONSTANTS (cm-1) ---

0.715143 0.012609 0.012421

Structure: Br-3-H-002

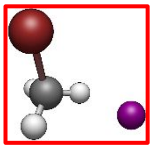

Charge: 1

Multiplicity: 3

Energy (Eh): -9761.09724498

ZPE (Eh): 0.02748675

E + ZPE (Eh): -9761.06975823

Rel. E (eV): 0.0497

SOCME (cm-1): 454.9508215181065

--- GEOMETRY (Angstroms) ---

C 1.471292 0.889654 0.027598

H 1.564148 1.950308 -0.163053

H 0.520540 0.358021 -0.137399

I -2.124972 -0.047931 -0.030060

H -2.151919 0.113410 1.589160

Br 2.971253 -0.154145 -0.000271

--- FREQUENCIES (cm-1) ---

33.03 55.9 87.98 138.33 242.39 360.35 713.8 834.09 1265.0 2249.38 2879.18 3205.87

--- ROTATIONAL CONSTANTS (cm-1) ---

0.926084 0.012834 0.012709

Structure: Br-H-SIDE_MECP

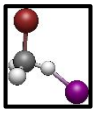

Charge: 1

Multiplicity:

Energy (Eh): -9761.13682297

ZPE (Eh): 0.03460250

E + ZPE (Eh): -9761.10222047

Rel. E (eV): -0.8337

SOCME (cm-1): 3002.4847110351784

--- GEOMETRY (Angstroms) ---

C -4.695813 -1.381877 -0.006948

H -4.887645 -1.926533 0.912026

H -4.904352 -1.914473 -0.929263

H -3.503178 -1.217215 -0.015294

Br -5.420182 0.337494 0.011084

I -1.536753 -1.749132 -0.050676

--- FREQUENCIES (cm-1) ---

26.08 210.64 274.83 700.67 957.75 959.33 1168.07 1227.42 1404.9 1970.2 3092.56 3196.28

--- ROTATIONAL CONSTANTS (cm-1) ---

0.745346 0.017280 0.016947

Structure: Br-1-TS-Brside_Hside

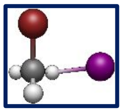

Charge: 1

Multiplicity: 1

Energy (Eh): -9761.10759646

ZPE (Eh): 0.03258908

E + ZPE (Eh): -9761.07500738

Rel. E (eV): -0.0932

SOCME (cm-1): 2920.827281781653

--- GEOMETRY (Angstroms) ---

C -1.292169 1.323017 0.004290

H -1.138952 1.649018 -1.015456

H -1.950468 1.965520 0.586866

H -0.278869 1.383047 0.562157

I 1.330955 0.186572 -0.027893

Br -1.904722 -0.530106 0.032662

--- FREQUENCIES (cm-1) ---

-2365.62 66.79 263.68 465.1 756.48 916.82 1136.61 1301.41 1417.46 1682.23 3086.79 3211.58

--- ROTATIONAL CONSTANTS (cm-1) ---

0.387939 0.030293 0.028259

Structure: IBr

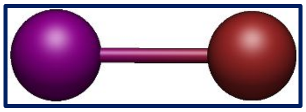

Charge: 0

Multiplicity: 1

Energy (Eh): -9721.61516968

ZPE (Eh): 0.00059329

E + ZPE (Eh): -9721.61457639

SOCME (cm-1): 1545.1928682206633

--- GEOMETRY (Angstroms) ---

I 0.000000 0.000000 0.995666

Br 0.000000 0.000000 -1.507121

--- FREQUENCIES (cm-1) ---

260.42

--- ROTATIONAL CONSTANTS (cm-1) ---

0.000000 0.054890 0.054890

Structure: IBr+

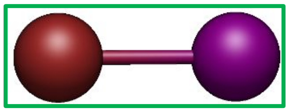

Charge: 1

Multiplicity: 2

Energy (Eh): -9721.25514375

ZPE (Eh): 0.00067810

E + ZPE (Eh): -9721.25446565

SOCME (cm-1): NaN

--- GEOMETRY (Angstroms) ---

I 0.000000 0.000000 0.959339

Br 0.000000 0.000000 -1.452281

--- FREQUENCIES (cm-1) ---

297.65

--- ROTATIONAL CONSTANTS (cm-1) ---

0.000000 0.059118 0.059118

Structure: CH3Br

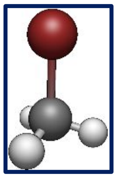

Charge: 0

Multiplicity: 1

Energy (Eh): -2646.06592035

ZPE (Eh): 0.03700432

E + ZPE (Eh): -2646.02891603

SOCME (cm-1): 1010.14644482895

--- GEOMETRY (Angstroms) ---

C -0.000000 -0.000000 -1.534982

H -0.000000 1.032790 -1.863203

H 0.894419 -0.516399 -1.863221

H -0.894419 -0.516399 -1.863221

Br 0.000000 0.000008 0.423061

--- FREQUENCIES (cm-1) ---

593.92 958.31 958.5 1326.9 1475.43 1475.51 3081.5 3186.36 3186.6

--- ROTATIONAL CONSTANTS (cm-1) ---

5.226266 0.313842 0.313842

Structure: CH3Br+

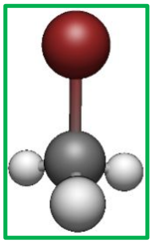

Charge: 1

Multiplicity: 2

Energy (Eh): -2645.68152701

ZPE (Eh): 0.03375065

E + ZPE (Eh): -2645.64777636

SOCME (cm-1): NaN

--- GEOMETRY (Angstroms) ---

C -1.525216 -0.000016 0.011672

H -1.768294 0.001270 -1.062793

H -1.844948 -0.925853 0.481400

H -1.844818 0.924918 0.483325

Br 0.417224 -0.000038 0.000491

--- FREQUENCIES (cm-1) ---

458.95 499.7 830.78 1188.12 1273.08 1408.93 2949.05 3099.53 3106.68

--- ROTATIONAL CONSTANTS (cm-1) ---

5.054190 0.320983 0.320268

80 0.376163 0.361406

Structure: CH2Br+

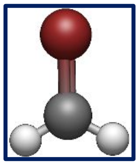

Charge: 1

Multiplicity: 1

Energy (Eh): -2645.08496651

ZPE (Eh): 0.02439403

E + ZPE (Eh): -2645.06057248

SOCME (cm-1): 993.0000000001302

--- GEOMETRY (Angstroms) ---

C -0.000001 0.000000 -1.400991

Br 0.000000 -0.000000 0.348483

H 0.000000 0.947624 -1.933051

H 0.000000 -0.947624 -1.933050

--- FREQUENCIES (cm-1) ---

862.74 971.67 1094.56 1444.47 3096.94 3237.36

--- ROTATIONAL CONSTANTS (cm-1) ---

9.311818 0.418600 0.400592

Structure: CH2Br+

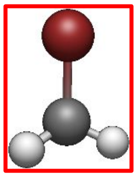

Charge: 1

Multiplicity: 3

Energy (Eh): -2644.99820870

ZPE (Eh): 0.02099078

E + ZPE (Eh): -2644.97721792

SOCME (cm-1): 1015.5294185857109

--- GEOMETRY (Angstroms) ---

C -0.018086 1.487015 0.000001

Br -0.011392 -0.364295 -0.000001

H 0.267071 1.929679 0.957140

H 0.267071 1.929678 -0.957140

--- FREQUENCIES (cm-1) ---

522.24 601.3 723.2 1226.95 3000.9 3139.29

--- ROTATIONAL CONSTANTS (cm-1) ---

8.520507 0.380984 0.366766

Structure: HBr

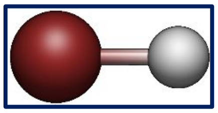

Charge: 0

Multiplicity: 1

Energy (Eh): -2606.75404320

ZPE (Eh): 0.00593476

E + ZPE (Eh): -2606.74810844

SOCME (cm-1): 991.2865377881412

--- GEOMETRY (Angstroms) ---

Br 0.000000 0.000000 0.038416

H 0.000000 0.000000 -1.384155

--- FREQUENCIES (cm-1) ---

2605.06

--- ROTATIONAL CONSTANTS (cm-1) ---

0.000000 8.368219 8.368219

Structure: Br

Charge: 0

Multiplicity: 2

Energy (Eh): -2606.10990244

ZPE (Eh): 0.00000000

E + ZPE (Eh): -2606.10990244

SOCME (cm-1): 0.0

--- GEOMETRY (Angstroms) ---

Br 0.000000 0.000000 0.000000

CH3I Reference E+ZPE (Hartrees): -14270.37615029

Structure: I-1-I-001

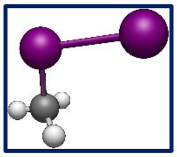

Charge: 1

Multiplicity: 1

Energy (Eh): -14270.52426815

ZPE (Eh): 0.03729386

E + ZPE (Eh): -14270.48697429

Rel. E (eV): -3.0157

SOCME (cm-1): 1399.6985426883891

--- GEOMETRY (Angstroms) ---

C -1.922062 1.657131 -0.000025

H -1.554842 2.114179 -0.910792

H -3.000677 1.519191 -0.000059

H -1.554887 2.114063 0.910820

I -1.160491 -0.384789 -0.000136

I 1.495742 0.086825 0.000142

--- FREQUENCIES (cm-1) ---

108.77 119.36 203.93 455.32 913.93 915.29 1283.95 1433.76 1446.38 3070.98 3192.89 3225.56

--- ROTATIONAL CONSTANTS (cm-1) ---

0.263877 0.032705 0.029267

Structure: I-3-I-001

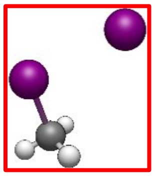

Charge: 1

Multiplicity: 3

Energy (Eh): -14270.49117272

ZPE (Eh): 0.03669299

E + ZPE (Eh): -14270.45447973

Rel. E (eV): -2.1315

SOCME (cm-1): 2274.9065916421623

--- GEOMETRY (Angstroms) ---

C -2.131360 1.671661 -0.000001

H -1.254694 2.309058 -0.000670

H -2.719932 1.766697 -0.905135

H -2.718836 1.767055 0.905804

I -1.399228 -0.374697 -0.000086

I 1.769769 0.073059 0.000086

--- FREQUENCIES (cm-1) ---

-37.47 63.51 103.61 473.07 895.33 903.4 1281.85 1443.72 1445.37 3080.41 3206.87 3209.21

--- ROTATIONAL CONSTANTS (cm-1) ---

0.265063 0.023445 0.021631

Structure: I-I-SIDE_MECP

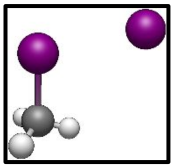

Charge: 1

Multiplicity: 3

Energy (Eh): -14270.49042137

ZPE (Eh): 0.03662070

E + ZPE (Eh): -14270.45380067

Rel. E (eV): -2.1130

SOCME (cm-1): 2459.3283640552677

--- GEOMETRY (Angstroms) ---

C -2.154905 1.677241 -0.000000

H -1.277641 2.313987 -0.000670

H -2.743109 1.777024 -0.904686

H -2.742015 1.777378 0.905353

I -1.427354 -0.368301 -0.000087

I 1.890742 0.035504 0.000088

--- FREQUENCIES (cm-1) ---

-58.76 58.76 78.16 475.96 890.58 903.63 1282.19 1443.07 1446.03 3081.3 3205.28 3209.66

--- ROTATIONAL CONSTANTS (cm-1) ---

0.267507 0.021489 0.019969

Structure: I-1-H-001

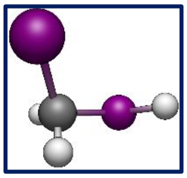

Charge: 1

Multiplicity: 1

Energy (Eh): -14270.48758908

ZPE (Eh): 0.03315591

E + ZPE (Eh): -14270.45443317

Rel. E (eV): -2.1302

SOCME (cm-1): 1429.3316453503714

--- GEOMETRY (Angstroms) ---

C 0.138613 1.076102 0.110961

H 0.017020 1.731506 -0.743642

H 0.024319 1.573587 1.065053

I 1.858462 -0.098130 -0.006430

H -1.493271 -1.045022 1.226053

I -1.853749 -0.063809 -0.022709

--- FREQUENCIES (cm-1) ---

95.57 125.24 321.36 521.53 576.59 750.8 1041.12 1140.18 1395.34 2238.88 3121.18 3225.98

--- ROTATIONAL CONSTANTS (cm-1) ---

0.659505 0.019150 0.018750

Structure: I-3-H-002

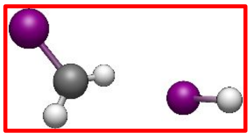

Charge: 1

Multiplicity: 3

Energy (Eh): -14270.41690322

ZPE (Eh): 0.02697278

E + ZPE (Eh): -14270.38993044

Rel. E (eV): -0.3750

SOCME (cm-1): 314.7413064724743

--- GEOMETRY (Angstroms) ---

C 1.062634 0.925166 0.016153

H 1.091917 1.988644 -0.170735

H 0.147774 0.336169 -0.027075

I -2.903869 -0.049787 -0.029189

H -3.106956 0.112861 1.573969

I 2.815824 -0.104612 0.002344

--- FREQUENCIES (cm-1) ---

12.96 40.49 56.0 102.84 168.5 231.49 613.64 774.91 1286.79 2265.02 3042.5 3244.54

--- ROTATIONAL CONSTANTS (cm-1) ---

0.908575 0.008014 0.007964

Structure: I-H-SIDE_MECP

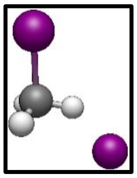

Charge: 1

Multiplicity:

Energy (Eh): -14270.45677197

ZPE (Eh): 0.03519544

E + ZPE (Eh): -14270.42157653

Rel. E (eV): -1.2361

SOCME (cm-1): 2898.637611016596

--- GEOMETRY (Angstroms) ---

C 8.468293 -0.738086 -0.072937

H 8.167801 -0.269420 -1.082236

H 8.315422 -1.808496 -0.145205

H 7.928316 -0.251241 0.730364

I 6.379945 0.222021 -2.242569

I 10.513216 -0.292779 0.054357

--- FREQUENCIES (cm-1) ---

-24.27 121.78 233.93 580.43 906.64 941.53 1216.43 1313.65 1405.29 2404.15 3116.66 3208.51

--- ROTATIONAL CONSTANTS (cm-1) ---

0.819540 0.011692 0.011555

Structure: I-3-TS-Iside_Hside

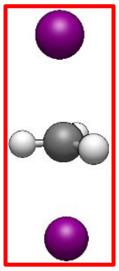

Charge: 1

Multiplicity: 3

Energy (Eh): -14270.44558931

ZPE (Eh): 0.03438130

E + ZPE (Eh): -14270.41120801

Rel. E (eV): -0.9540

SOCME (cm-1): 13.654237437513675

--- GEOMETRY (Angstroms) ---

C 0.649999 1.309544 0.034920

H 0.983926 1.779046 0.945144

H 0.858655 1.769977 -0.915715

H 0.109282 0.379166 0.077275

I -1.687474 2.680583 0.203898

I 2.993397 -0.057101 -0.143481

--- FREQUENCIES (cm-1) ---

-224.42 86.16 114.05 123.82 405.54 874.85 1030.85 1321.68 1397.73 3124.06 3301.08 3311.82

--- ROTATIONAL CONSTANTS (cm-1) ---

4.805962 0.008990 0.008990

Structure: I-1-Iside_Hside

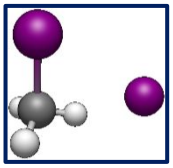

Charge: 1

Multiplicity: 1

Energy (Eh): -14270.43551270

ZPE (Eh): 0.03280186

E + ZPE (Eh): -14270.40271084

Rel. E (eV): -0.7227

SOCME (cm-1): 2893.0618797391803

--- GEOMETRY (Angstroms) ---

C 0.974986 1.528896 0.027194

H 1.203813 2.070682 -0.884329

H 1.167680 2.058574 0.954506

I 1.886218 -0.344402 0.031371

I -1.951641 -0.087989 -0.130486

H -0.140119 1.242678 0.006451

--- FREQUENCIES (cm-1) ---

-654.86 58.18 239.3 526.32 747.27 895.19 1156.72 1225.71 1394.65 1846.51 3103.95 3204.54

--- ROTATIONAL CONSTANTS (cm-1) ---

0.331566 0.017671 0.016834

Structure: I2

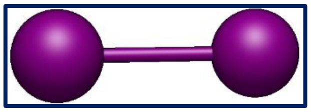

Charge: 0

Multiplicity: 1

Energy (Eh): -14230.93000675

ZPE (Eh): 0.00047885

E + ZPE (Eh): -14230.92952790

SOCME (cm-1): 7.788530028188888e-05

--- GEOMETRY (Angstroms) ---

I 0.000000 0.000000 1.352151

I 0.000000 0.000000 -1.352151

--- FREQUENCIES (cm-1) ---

210.19

--- ROTATIONAL CONSTANTS (cm-1) ---

0.000000 0.036329 0.036329

Structure: I2+

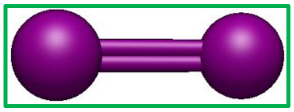

Charge: 1

Multiplicity: 2

Energy (Eh): -14230.58566801

ZPE (Eh): 0.00053997

E + ZPE (Eh): -14230.58512804

SOCME (cm-1): NaN

--- GEOMETRY (Angstroms) ---

I 0.000000 0.000000 1.309079

I 0.000000 0.000000 -1.309079

--- FREQUENCIES (cm-1) ---

237.02

--- ROTATIONAL CONSTANTS (cm-1) ---

0.000000 0.038759 0.038759

Structure: CH3I

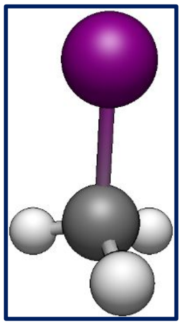

Charge: 0

Multiplicity: 1

Energy (Eh): -7155.36992276

ZPE (Eh): 0.03644026

E + ZPE (Eh): -7155.33348250

SOCME (cm-1): 2133.3215885097766

--- GEOMETRY (Angstroms) ---

C 0.000000 0.000003 -1.828778

H -0.000000 1.033396 -2.153516

H 0.894947 -0.516700 -2.153519

H -0.894946 -0.516700 -2.153519

I -0.000000 0.000001 0.331066

--- FREQUENCIES (cm-1) ---

525.68 890.5 890.7 1275.78 1469.95 1469.98 3085.21 3193.71 3193.92

--- ROTATIONAL CONSTANTS (cm-1) ---

5.220131 0.246359 0.246359

Structure: CH3I+

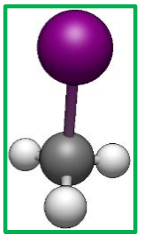

Charge: 1

Multiplicity: 2

Energy (Eh): -7155.01610075

ZPE (Eh): 0.03322698

E + ZPE (Eh): -7154.98287377

SOCME (cm-1): NaN

--- GEOMETRY (Angstroms) ---

C -2.112949 1.676220 0.000045

H -1.242814 2.320050 -0.000713

H -2.727793 1.751714 -0.896354

H -2.726787 1.752147 0.897058

I -1.401063 -0.357934 -0.000124

--- FREQUENCIES (cm-1) ---

-407.9 457.9 919.46 1249.59 1271.59 1415.68 3016.32 3038.36 3216.06

--- ROTATIONAL CONSTANTS (cm-1) ---

5.079073 0.249076 0.248793

Structure: CH2I+

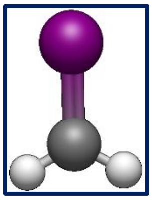

Charge: 1

Multiplicity: 1

Energy (Eh): -7154.39814233

ZPE (Eh): 0.02373511

E + ZPE (Eh): -7154.37440722

SOCME (cm-1): 2115.6748332387942

--- GEOMETRY (Angstroms) ---

C -1.927250 1.551783 -0.262396

H -1.295300 2.347900 0.117666

H -2.827517 1.787808 -0.820455

I -1.443489 -0.310086 0.059191

--- FREQUENCIES (cm-1) ---

749.5 878.65 1017.3 1414.7 3110.71 3247.65

--- ROTATIONAL CONSTANTS (cm-1) ---

9.444609 0.321364 0.310789

Structure: CH2I+

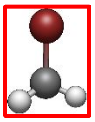

Charge: 1

Multiplicity: 3

Energy (Eh): -7154.33523770

ZPE (Eh): 0.02114083

E + ZPE (Eh): -7154.31409687

SOCME (cm-1): 2142.0168066567544

--- GEOMETRY (Angstroms) ---

C -2.012357 1.606090 -0.157014

H -1.255014 2.344325 0.088262

H -2.811403 1.772966 -0.872834

I -1.414781 -0.345976 0.035592

--- FREQUENCIES (cm-1) ---

411.94 561.62 734.33 1268.3 3074.3 3229.26

--- ROTATIONAL CONSTANTS (cm-1) ---

8.728744 0.295517 0.286621

Structure: I

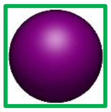

Charge: 0

Multiplicity: 2

Energy (Eh): -7115.43074969

ZPE (Eh): 0.00000000

E + ZPE (Eh): -7115.43074969

Rel. E (eV): 194696.0527

SOCME (cm-1): NaN

--- GEOMETRY (Angstroms) ---

I 0.000000 0.000000 0.000000

Structure: HI

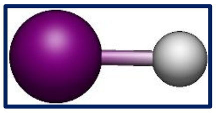

Charge: 0

Multiplicity: 1

Energy (Eh): -7116.05482380

ZPE (Eh): 0.00523376

E + ZPE (Eh): -7116.04959004

--- GEOMETRY (Angstroms) ---

I 0.000000 0.000000 0.030345

H 0.000000 0.000000 -1.586053

--- FREQUENCIES (cm-1) ---

2297.35

--- ROTATIONAL CONSTANTS (cm-1) ---

0.000000 6.451720 6.451720

Structure: HI

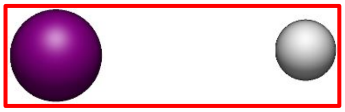

Charge: 0

Multiplicity: 3

Energy (Eh): -7115.92994653

ZPE (Eh): 0.00032580

E + ZPE (Eh): -7115.92962073

SOCME (cm-1): 0.0

--- GEOMETRY (Angstroms) ---

I 0.000000 0.000000 -0.104125

H 0.000000 0.000000 -3.197618

--- FREQUENCIES (cm-1) ---

143.01

--- ROTATIONAL CONSTANTS (cm-1) ---

0.000000 1.761466 1.761466

Structure: I+

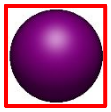

Charge: 1

Multiplicity: 3

Energy (Eh): -7115.04266779

ZPE (Eh): 0.00000000

E + ZPE (Eh): -7115.04266779

SOCME (cm-1): 3955.853384542961

--- GEOMETRY (Angstroms) ---

I 0.000000 0.000000 0.000000

Structure: I+

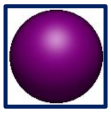

Charge: 1

Multiplicity: 1

Energy (Eh): -7114.98759387

ZPE (Eh): 0.00000000

E + ZPE (Eh): -7114.98759387

SOCME (cm-1): 3955.853384542961

--- GEOMETRY (Angstroms) ---

I 0.000000 0.000000 0.000000

Structure: CH3

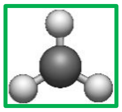

Charge: 0

Multiplicity: 2

Energy (Eh): -39.84281569

ZPE (Eh): 0.02968632

E + ZPE (Eh): -39.81312937

SOCME (cm-1): NaN

--- GEOMETRY (Angstroms) ---

C -0.000000 -0.000000 -0.000393

H 0.000000 1.078377 -0.000718

H -0.933916 -0.539189 -0.000718

H 0.933916 -0.539189 -0.000718

--- FREQUENCIES (cm-1) ---

534.87 1407.44 1407.6 3109.25 3285.72 3285.9

--- ROTATIONAL CONSTANTS (cm-1) ---

9.587459 9.587181 4.793661

Structure: CH3+

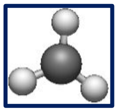

Charge: 1

Multiplicity: 1

Energy (Eh): -39.48214018

ZPE (Eh): 0.03119311

E + ZPE (Eh): -39.45094707

SOCME (cm-1): 17.94989972116836

--- GEOMETRY (Angstroms) ---

C 0.000024 -0.000051 -0.000001

H 0.150813 1.080647 0.000000

H 0.860336 -0.671277 0.000000

H -1.011172 -0.410156 0.000000

--- FREQUENCIES (cm-1) ---

1410.4 1410.91 1423.6 3021.42 3212.8 3213.08

--- ROTATIONAL CONSTANTS (cm-1) ---

9.367194 9.360368 4.681890

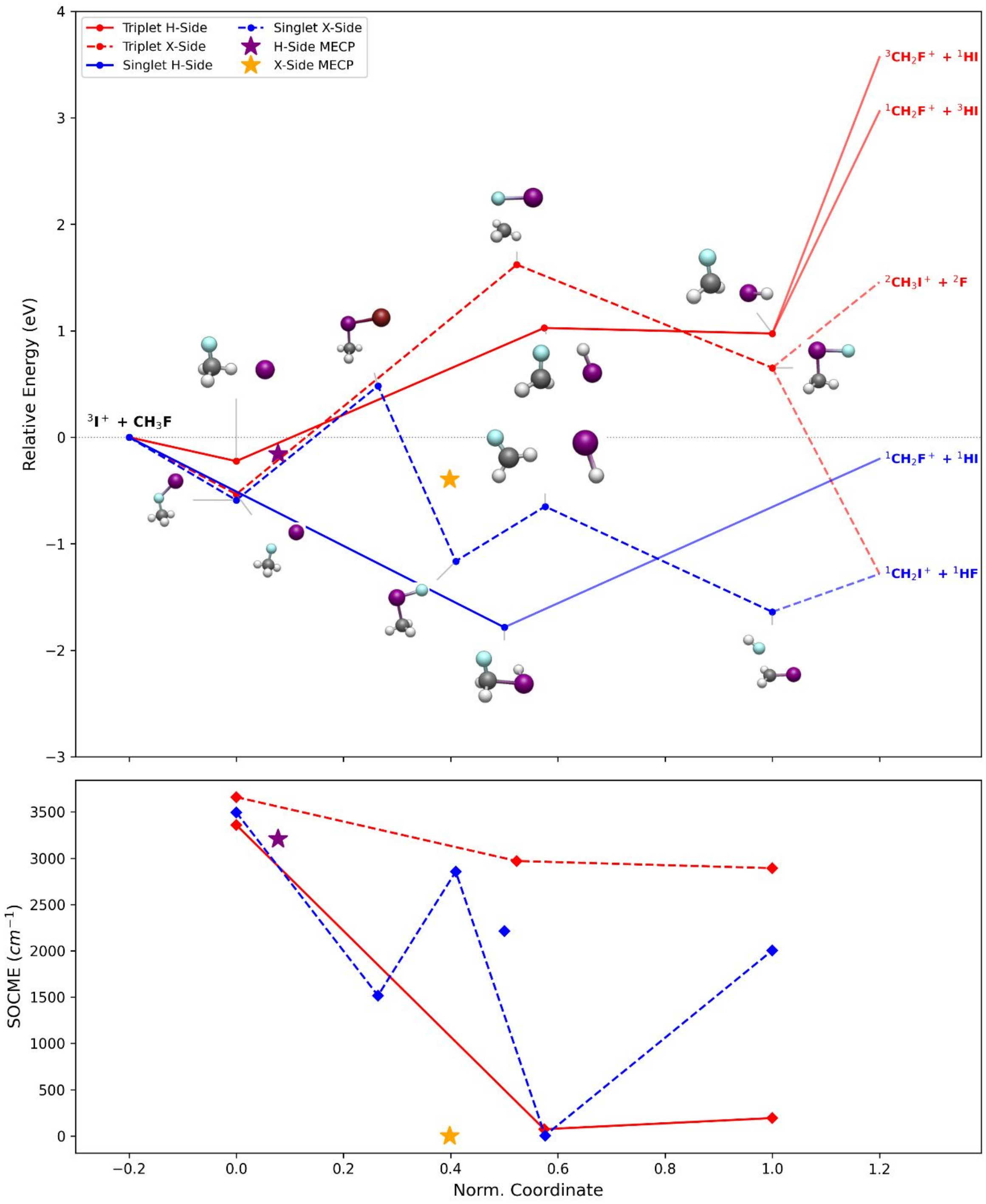

Figure S1. Calculated spin-orbit coupling matrix elements (SOCME) at TD-DFT B3LYP/x2c-TZVPPall (bottom panel) compared to reaction coordinate (top panel) for the $I^{+} + CH_3F$ reaction. The x-axis is the normalized reaction coordinate found by taking the RMSD difference between each structure and the product complex, divided by the total RMSD difference of the reactant complex and product complex. The separated reactants and products are plotted at -0.2 and 1.2 respectively for reference. SOCME values of 0 (The MECP's in this figure) are due to the TD-DFT calculations failing, and are shown for reference.

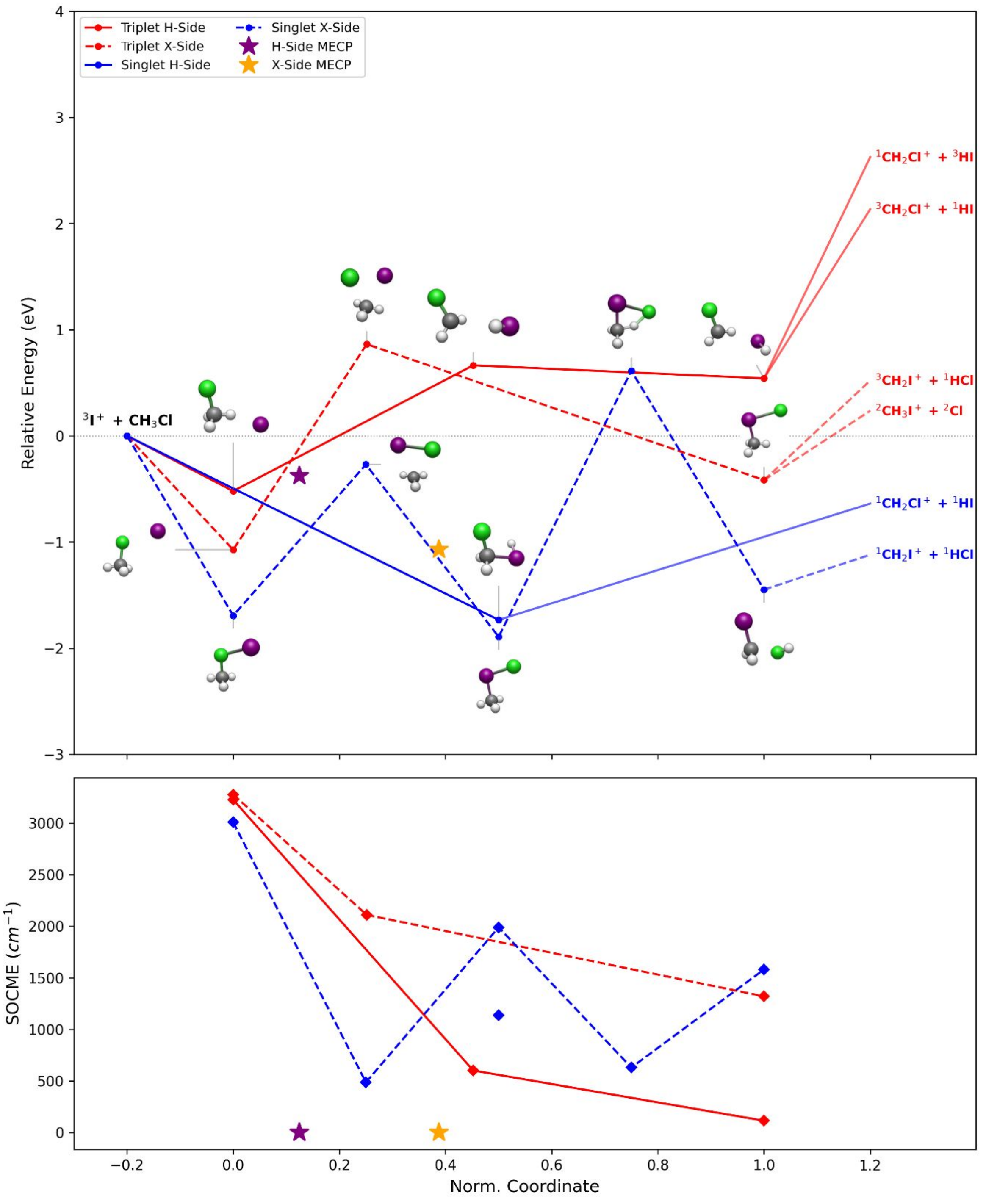

Figure S2. As Figure S1 but for the $I^+ + CH_3Cl$ reaction.

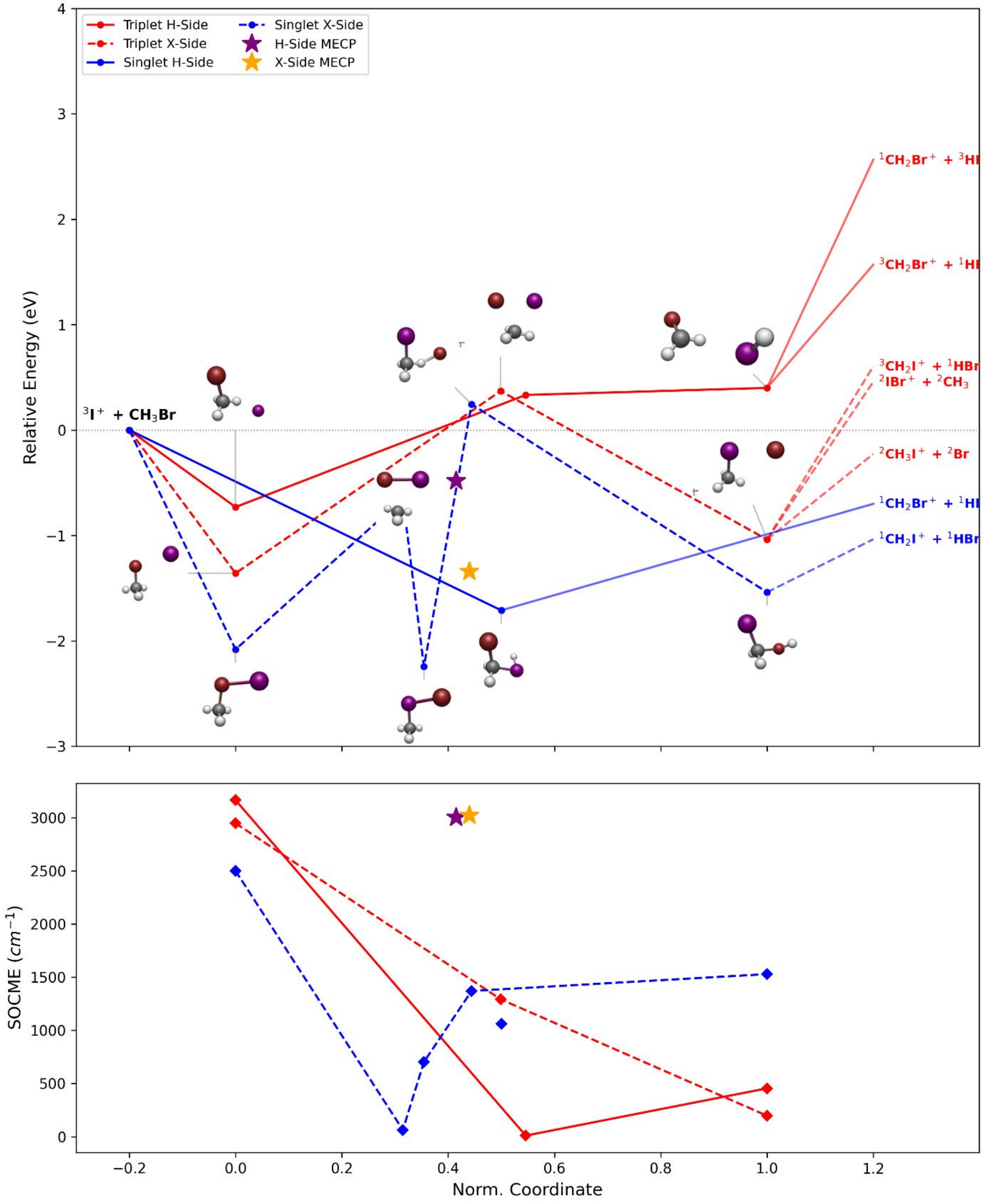

Figure S3. As Figure S1 but for the $I^+ + CH_3Br$ reaction

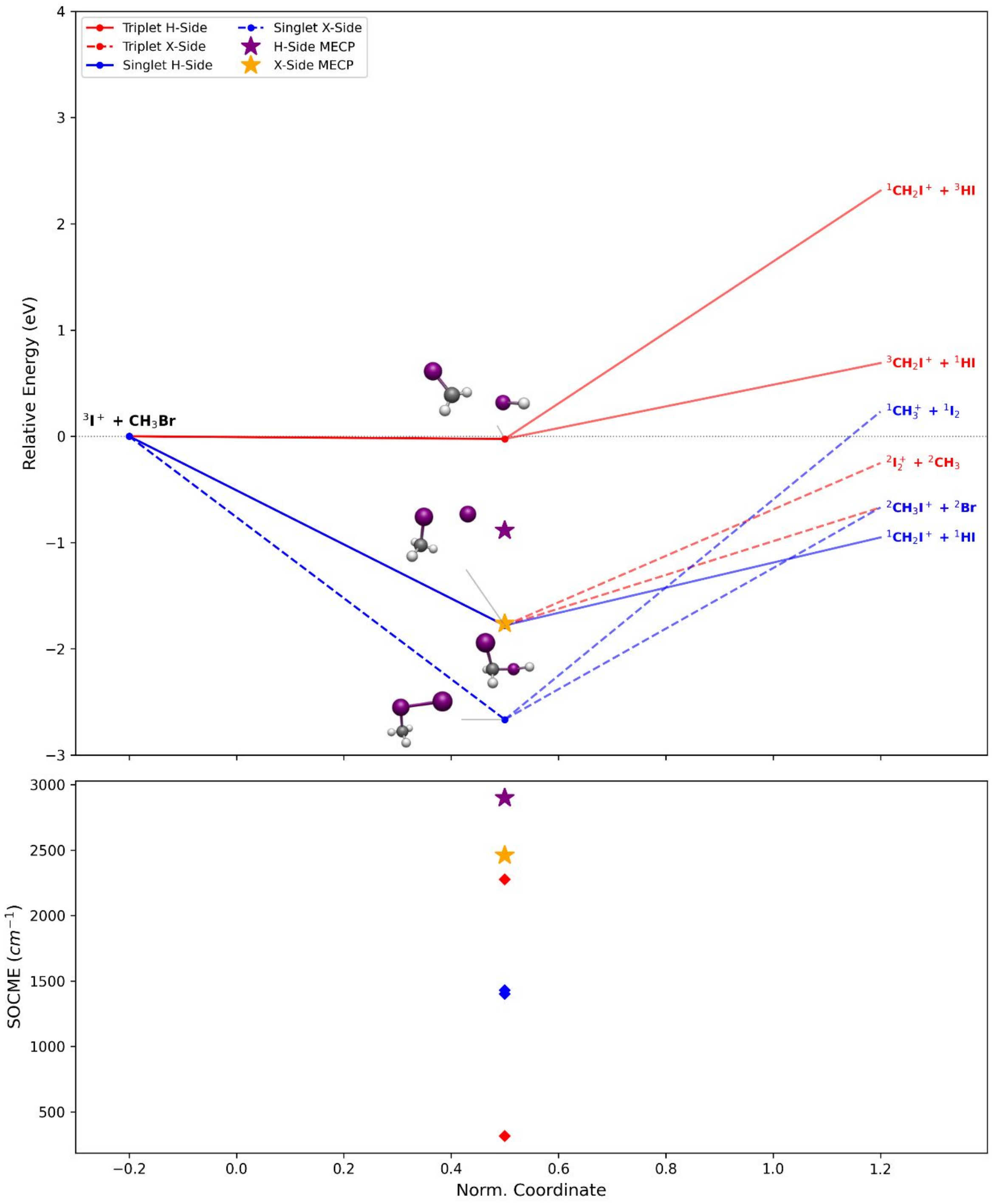

Figure S4. As Figure S1, but for the $I^+ + CH_3I$ reaction.
